# Loss of Gsx1 and Gsx2 Function Rescues Distinct Phenotypes in Dlx1/2 Mutants

**DOI:** 10.1002/cne.23242

**Published:** 2012-10-08

**Authors:** Bei Wang, Jason E Long, Pierre Flandin, Ramon Pla, Ronald R Waclaw, Kenneth Campbell, John LR Rubenstein

**Affiliations:** 1 Department of Psychiatry and the Nina Ireland Laboratory of Developmental Neurobiology, University of California San FranciscoSan Francisco, California 94158-2324; 2 Division of Developmental Biology, Cincinnati Children's Hospital Medical Center, University of Cincinnati College of MedicineCincinnati, Ohio 45229

**Keywords:** Dlx, Gsx, CGE, LGE, MGE, septum, development, mutant, interneuron, GABA, basal ganglia

## Abstract

Mice lacking the Dlx1 and Dlx2 homeobox genes (Dlx1/2 mutants) have severe deficits in subpallial differentiation, including overexpression of the Gsx1 and Gsx2 homeobox genes. To investigate whether Gsx overexpression contributes to the Dlx1/2 mutant phenotypes, we made compound loss-of-function mutants. Eliminating Gsx2 function from the Dlx1/2 mutants rescued the increased expression of Ascl1 and Hes5 (Notch signaling mediators) and Olig2 (oligodendrogenesis mediator). In addition, Dlx1/2;Gsx2 mutants, like Dlx1/2;Ascl1 mutants, exacerbated the Gsx2 and Dlx1/2 patterning and differentiation phenotypes, particularly in the lateral ganglionic eminence (LGE) caudal ganglionic eminence (CGE), and septum, including loss of GAD1 expression. On the other hand, eliminating Gsx1 function from the Dlx1/2 mutants (Dlx1/2;Gsx1 mutants) did not severely exacerbate their phenotype; on the contrary, it resulted in a partial rescue of medial ganglionic eminence (MGE) properties, including interneuron migration to the cortex. Thus, despite their redundant properties, Gsx1 and -2 have distinct interactions with Dlx1 and -2. Gsx2 interaction is strongest in the LGE, CGE, and septum, whereas the Gsx1 interaction is strongest in the MGE. From these studies, and earlier studies, we present a model of the transcriptional network that regulates early steps of subcortical development. J. Comp. Neurol. 521:1561–1584, 2013. © 2012 Wiley Periodicals, Inc.

Transcription programs that regulate the genetic circuits underlying regional and cell type identity in the forebrain are beginning to be elucidated. Here we focus on the development of mouse subcortical telencephalic domains, known as the lateral, medial, and caudal ganglionic eminences (LGE, MGE, and CGE, respectively), and the septum (Flames et al., 2007). These primorida generate γ-aminobutyric acid (GABA)-ergic projection neurons of subpallial nuclei (e.g., striatum and globus pallidus) and GABAergic interneurons of the pallium and olfactory bulb. Over 100 transcription factors have been implicated in mediating their development (Long et al., 2009a,b), including the Dlx1 and -2 and Gsx1 and -2 (Gsh1 and -2) homeobox genes. These transcription factors are expressed in primary and secondary progenitors in the ventricular and subventricular zones (VZ and SVZ, respectively); as neurons are produced, subsets continue to express Dlx1 and/or Dlx2 (Cobos et al., 2005a, 2007, and unpublished data). Gsx1 and -2 have partially redundant functions and together promote LGE regional fate (Corbin et al., 2000; Toresson et al., 2000; Toresson and Campbell, 2001; Yun et al., 2001, 2003; Waclaw et al., 2004), whereas Dlx1 and -2, which are linked genes, promote later steps in subcortical differentiation, in part through inducing the expression of Arx, Dlx5, and Dlx6 homeobox genes (except in the septum; Anderson et al., 1997a; Cobos et al., 2005b; Long et al., 2007, 2009a,b).

Different Dlx gene dosages control different processes. Dlx1 and -2 mutants lack expression of Dlx1/2/5/6; loss of expression of these eight Dlx alleles uncovers the fundamental Dlx-mediated programs, which include 1) repressing Notch signaling and glial differentiation, through decreasing Ascl1 (Mash1) and Olig2 expression (Yun et al., 2002; Petryniak et al., 2007); 2) promoting GABAergic neural differentiation through inducing expression of glutamic acid decarboxylase (GAD1 and -2) and vesicular GABA transporter (Anderson et al., 1997a; Long et al., 2009a,b); and 3) promoting neuronal migration through repressing neurite outgrowth and Pak3 kinase expression and by maintaining expression of Cxcr4 and -7 cytokine receptors (Anderson et al., 1997b; Long et al., 2007; Cobos et al., 2007; Wang et al., 2011). More subtle phenotypes arise in Dlx1^−/−^;Dlx2^+/−^ mutants, which have defects in synapse development (Stanco, Cobos, and Rubenstein, unpublished). Dlx1^−/−^ mutants show defects in survival of a subset of subcortically derived cortical interneurons (dendrite innervating interneurons; Cobos et al., 2005a). Loss of Dlx5 and -6 results in defects in interneuron migration and differentiation (Wang et al., 2010). Reduced Dlx dosage (Dlx1^−/−^, Dlx5/6^+/−^) results in viable mice that have abnormal cortical function, epilepsy, and fear behaviors (Cobos et al., 2005a; Mao et al., 2009, 2011; Wang et al., 2010).

The Gsx2 and Dlx1 and -2 genes mediate their subcortical transcriptional programs in combination with the Ascl1 (Mash1) bHLH gene. A feature of the Dlx1/2 mutants is overexpression of Ascl1, Gsx1, and Gsx2 (Yun et al., 2002; Long et al., 2009a,b). We hypothesized that some of the Dlx1/2 mutant phenotype is caused by the increased levels of Ascl1, Gsx1, and Gsx2 and therefore set out to make compound mutants that reduce Ascl1, Gsx1, or Gsx2 dosage in Dlx1/2 mutants. Dlx1/2;Ascl1 compound mutants do not exhibit a rescue of Dlx1/2 mutant properties; rather, their phenotype is much more severe than that of the individual mutants, because Dlx1/2 and Ascl1 regulate parallel pathways of subcortical differentiation (Long et al., 2009a,b).

Here we explored the effect of reducing Gsx1 or Gsx2 expression in the Dlx1/2 mutants by making compound mutants. We performed our phenotypic analysis on the CGE, LGE, MGE, and septum and focused on the expression of transcription factors that are abnormally expressed in the Dlx1/2 mutants (Long et al., 2009a,b). We also focused on GAD1 expression, given its fundamental importance in defining the GABAergic phenotype.

Eliminating Gsx2 function from the Dlx1/2 mutants rescued the increased expression of Hes5 (Notch signaling indicator), Olig2 (oligodendrogenesis indicator), and Gbx1 (unknown function). In addition, Dlx1/2;Gsx2 mutants, like Dlx1/2;Ascl1 mutants, showed an exacerbation of the Gsx2 and Dlx1/2 mutant phenotypes, including GAD1 expression, particularly in the LGE, CGE, and septum. On the other hand, eliminating Gsx1 function from the Dlx1/2 mutants (Dlx1/2;Gsx1 mutants) did not severely exacerbate their phenotype; rather, the mutants exhibited a partial rescue of MGE properties and MGE interneuron migration to the cortex. Thus, despite their partially redundant properties, Gsx1 and Gsx2 have distinct interactions with the Dlx1/2 mutants. We present a model of the transcriptional network that regulates early steps of subcortical development.

## MATERIALS AND METHODS

### Animals

Mice were maintained under standard conditions with food and water ad libitum. All experimental procedures were approved by the Committee on Animal Health and Care at the University of California, San Francisco (UCSF). Mouse colonies were maintained at UCSF, in accordance with National Institutes of Health and UCSF guidelines. Mouse strains with a null allele of Dlx1, Dlx2, Gsx1, and Gsx2 were used in this study (Anderson et al., 1997b; Casarosa et al., 1999). These strains were maintained on a CD-1 background. For staging of embryos, midday of the vaginal plug was calculated as embryonic day 0.5 (E0.5). PCR genotyping was performed as described elsewhere (Anderson et al., 1997b; Casarosa et al., 1999). Gsx1 and -2 genotyping was performed as described by Yun et al. (2003) and Wang et al. (2009).

### Tissue preparation, in situ hybridization, and immunofluoresence

Preparation of sectioned embryos, immunofluoresence, and in situ hybridization were performed using digoxigenin riboprobes on 20-μm frozen sections cut on a cryostat using methods described by Long et al. (2007, 2009a,b). We used a rabbit polyclonal anti-GSX2 antibody (Toresson et al., 2000), a guinea pig polyclonal anti-DLX2 (Kuwajima et al., 2006), and a mouse monoclonal anti-MASH1 (BD Pharmingen, San Jose, CA) Riboprobes have been described by Long et al. (2009a,b), except for Ngn2 (1.5-kb mouse full coding sequence from Francois Guillemot).

### Antibody specificity characterization

DLX2 immunoreactivity closely matches endogenous Dlx2 RNA expression and disappears in the brain of Dlx1/2^−/−^ mutants (Long et al., 2007). In Dlx1/2 mutants, a deletion removes most of the coding exons for both Dlx1 and Dlx2 but does not remove exon 1, so an N-terminal truncated protein could be produced. Because the guinea pig anti-DLX2 antibody was made to the N-terminal amino acids (1–154; Kuwajima et al., 2006), we conclude that very little of this protein is present in the Dlx1/2^−/−^ mutant brain (Table 1).

**TABLE 1:** Antibody Characterization

Antigen	Immunogen	Species; manufacturer	Dilution
ASCL1 (MASH)	Full-length ASCL1	Mouse monoclonal; BD Pharmigen; catalog No. 556604	1/500
DLX2	N-terminal amino acids 1–154	Guinea pig; Kuwajima et al. (2006)	1/3,000
GSX2	10-mer peptide (ANEDKEISPL) from the C-terminal of the protein	Rabbit; Pei et al. (2011)	1/500

GSX2 immunoreactivity closely matches endogenous Gsx2 RNA expression and disappears in the brain of Gsx2^−/−^ mutants (Toresson et al., 2000). Moreover, it does not recognize GSX1 even when Gsx1 is overexpressed (Pei et al., 2011).

ASCL1 antibody specificity was tested using lysate from rat embryonic brain by Western blot; the antibody specifically recognized a 34-kDa protein (information provided by the manufacturer; BD Pharmingen product 556004). Furthermore, its immunoreactivity in the sections from the embryonic mouse brain closely resembles the expression of ASCL1 RNA, as detected here by in situ hybridization.

### Microscopy

Images of in situ hybridization results were captured with a Zeiss AxioCam MR (Zeiss, Thornwood, NY) and saved as TIFF files. Images of immunofluoresence were captured with a Zeiss LSM 510 confocal microscope. The images were then processed in Adobe Photoshop CS3 to optimize the contrast and brightness to illustrate best the gene expression patterns.

### Analysis of the number of DLX2-, GSX2-, and ASCL1-expressing cells

**Figure 1:**
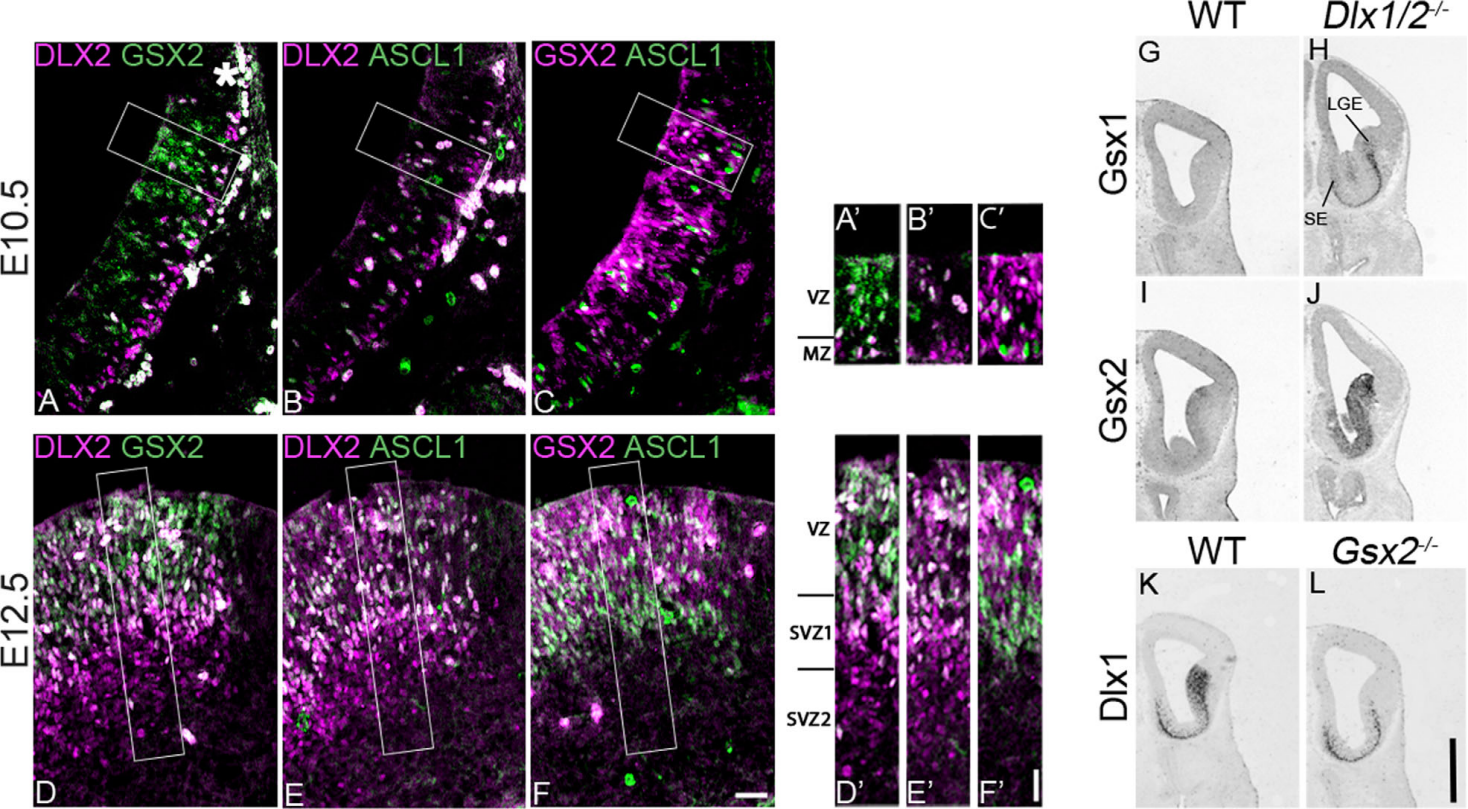
LGE expression of ASCL1, DLX2, and GSX2 proteins using two-color immunofluoresence, at E10.5 (**A–C**) and E12.5 (**D–F**). Cells (nuclei) expressing both proteins are yellow; green and red correspond to specific transcription factors defined at the top of each panel. The boxed areas in each panel are shown at higher magnification in **A′–F**′. E12.5 data showing previously published information about the relationship between Gsx and Dlx expression in Gsx2 and Dlx1/2 mutants. Dlx1/2 mutants overexpress Gsx1 (**G,H**) and Gsx2 (**I,J**). Gsx2 mutants express less Dlx1, especially in the dorsal LGE (**K,L**). LGE, lateral ganglionic eminence; MZ, mantle zone; SE, septum; SVZ1 and SVZ2, subventricular zones 1 and 2. Scale bars = 20 μm in F (applies to A–F); 20 μm in F′ (applies to A′–F′); 500 μm in L (applies to G–L).

DLX2-, GSX2-, and MASH1-expressing cells (nuclei) were visualized on 20-μm coronal forebrain sections from E10.5 and E12.5 wild-type mice by immunofluorescence confocal microscopy. The images were imported into Adobe Photoshop CS3, and a rectangle encompassing the VZ and SVZ domains was placed orthogonal to the ventricular surface (see Fig. 1). The labeled cells in the VZ, SVZ1, and SVZ2 within each rectangle were manually counted from one section by using the Photoshop counting tool. We calculated the mean size of the stained part of the cell (nucleus) separately for each population of neurons we counted, and at each age, and then corrected the profile counts separately for each population using the Abercrombie equation with the mean nuclear diameter for that population (Guillery, 2002). The average size of the staining was as follows at E10.5: Dlx2, 4.3 ± 0.2 μm (SEM); Gsx2,. 4.2 ± 0.2 μm; Ascl1, 4.0 ± 0.2 μm; and, at E12.5: Dlx2, 4.3 ± 0.2 μm (SEM); Gsx2, 4.5 ± 0.2 μm; Ascl1, 4.0 ± 0.2 μm. For Table 2, we used the following scoring system to describe the number of positive nuclei/section: 1+ = 1–9, 2+ = 10–29, 3+ = 30–59, 4+ = ≥60.

**TABLE 2:** Cells Expressing Ascl1, Dlx2, and Gsx2 in the E10.5 and E12.5 Dorsal LGE^1^

	E10.5		E12.5		
		
	VZ	MZ	VZ	SVZ1	SVZ2
Dlx2	2+	1+	4+	3+	3+
Gsx2	2+	1+	4+	3+	1+
Dlx2/Gsx2	2+	1+	4+	3+	1+
Dlx2	2+	1+	4+	3+	3+
Ascl1	2+	1+	3+	3+	1+
Dlx2/Ascl1	1+	1+	3+	3+	1+
Gsx2	2+	1+	—	—	1+
Ascl1	1+	1+	4+	3+	1+
Gsx2/Ascl1	1+	1+	3+	3+	1+

1 See data in Figure 1. A dash indicates that Gsx2^+^ cell density was too high to count with accuracy but clearly was greater than Ascl1 density. The scoring system for positive nuclei/section is 1+ = 1–9, 2+ = 10–29, 3+ = 30–59, 4+ = ≥60.

### Qualitative analysis of gene expression changes

In describing the gene expression changes between control and mutant brains, in the text and in Figures 16 and 17, we made our best judgment assessments, independently by two or three people during at least two separate rating sessions. Similar approaches and figures were used by Long et al. (2009a,b) in describing gene expression changes in the Dlx1/2^−/−^, Ascl1^−/−^, and Dlx1/2;Ascl1^−/−^ mutants, so we have used a related system in this paper to allow comparison between these mutants. In Figures 16 and 17, we used a color-coded scoring system in which black indicates that expression was not analyzed (if no squares are listed, this also means that this analysis was not performed); gray indicates that expression was not clearly changed in the mutant; white indicates no detectable expression; red indicates severe reduction in expression; orange indicates moderate/mild reduction in expression; green indicates ectopic expression; and blue indicates increased expression. If the box is subdivided diagonally, the top part corresponds to the dorsal region, the bottom to the ventral region.

## RESULTS

### Subpallial expression of DLX2, GSX2, and ASCL1 proteins

To compare DLX2, GSX2, and ASCL1 protein expression at the cellular level in the developing subpallium, we used double immunofluorescence at E10.5, E12.5, and E15.5 (Fig. 1A–F, and not shown). We focused on expression in the rostral telencephalon in the region of the LGE and septum. These analyses complement previous studies that examined expression of GSX2 (Corbin et al., 2000; Yun et al., 2003; Wang et al., 2009), DLX2 (Porteus et al., 1994; Eisenstat et al., 1999; Yun et al., 2002), DLX2 and ASCL1 (Porteus et al., 1994; Yun et al., 2002), DLX2 and OLIG2, and ASCL1 and OLIG2 (Petryniak et al., 2007).

At E10.5, most VZ progenitors in the dorsal LGE (dLGE) strongly expressed GSX2, with weaker expression spreading ventrally through the ventral LGE (vLGE) and the MGE (Fig. 1A–C, and not shown). ASCL1^+^ cells were scattered throughout the VZ and mantle zone (MZ) of the LGE and MGE. DLX2^+^ cells in the VZ were most concentrated in the dLGE, whereas the thin MZ had many DLX2^+^ cells. In the VZ (of the LGE), most ASCL1^+^ and DLX2^+^ cells coexpress GSX2, whereas, in the MZ, most ASCL1^+^ and DLX2^+^ cells were GSX2^−^ (Table 1).

Expression at E12.5 and E15.5 showed similar results (Fig. 1D–F, and not shown). The main difference was the formation of the SVZ. Previously, we presented evidence that the SVZ consists of two layers, SVZ1 (adjacent to the VZ) and SVZ2 (Yun et al., 2002; Petryniak et al., 2007). DLX2 was robustly expressed in nearly all cells in SVZ1 and SVZ2. The intensity of GSX2 expression decreased as cells moved from the VZ to SVZ1, although most cells continued to express detectable GSX2 and coexpressed DLX2 and ASCL1 (Table 1). However, in SVZ2, GSX2 and ASCL1 expression was at background levels, except in occasional cells.

Analysis of GSX1 protein expression was hampered by the lack of a specific antibody. However, previous analyses showed that expression of Gsx1 RNA, and EGFP from a BAC transgenic, is largely complementary to that of Gsx2 at E12.5 and later stages. Whereas Gsx2 was highly expressed in the VZ, Gsx1 expression began in SVZ1. Furthermore, although their expression overlapped along the dorsoventral axis of the subpallium, Gsx2 was most strongly expressed in the LGE, CGE, and septum, and Gsx1 was most strongly expressed in the MGE (Toresson et al., 2000; Yun et al., 2001; Pei et al., 2011). Overall, GSX2 expression in the VZ was temporally upstream of DLX2 expression; as progenitors mature to the SVZ1 state, there generally was coexpression of GSX2, ASCL1, and DLX2. We used analysis of Gsx2^−/−^;Dlx1/2^−/−^ (Gsx2;Dlx1/2) compound mutants to assess the effects of losing expression of these transcription factors in the same progenitor cells.

### Combined functions of Gsx2 and Dlx1/2 in defining regional identity of LGE and CGE progenitor cells

Previous studies showed that Gsx2 promoted the expression of the Dlx genes (Corbin et al., 2000; Toresson et al., 2000, 2001; Yun et al., 2001, 2003), whereas Dlx1/2 repressed Gsx1 and Gsx2 expression (Fig. 1G–L; Yun et al., 2002; Long et al., 2009a). Both Gsx2 and Dlx1/2 promoted LGE (dLGE) identity. Loss of Gsx2 resulted in transformation of the dLGE toward a ventral pallial fate (Corbin et al., 2000; Toresson et al, 2000; Toresson and Campbell, 2001; Yun et al., 2001, 2003; Waclaw et al., 2004); loss of Dlx1/2 transformed neurons in the rostrodorsal striatal region toward mixed pallial/subpallial properties (Long et al., 2009a).

Here we further investigated Gsx2 function, with the goal of understanding the ramifications of its upregulation in the Dlx1/2 mutant SVZ (Long et al., 2009a,b). We studied the phenotype of the Gsx2;Dlx1/2 compound mutants to determine 1) the combined functions of Dlx1/2 and Gsx2 and 2) whether some of the Dlx1/2 mutant phenotype was reversed by removing expression of Gsx2.

**Figure 2:**
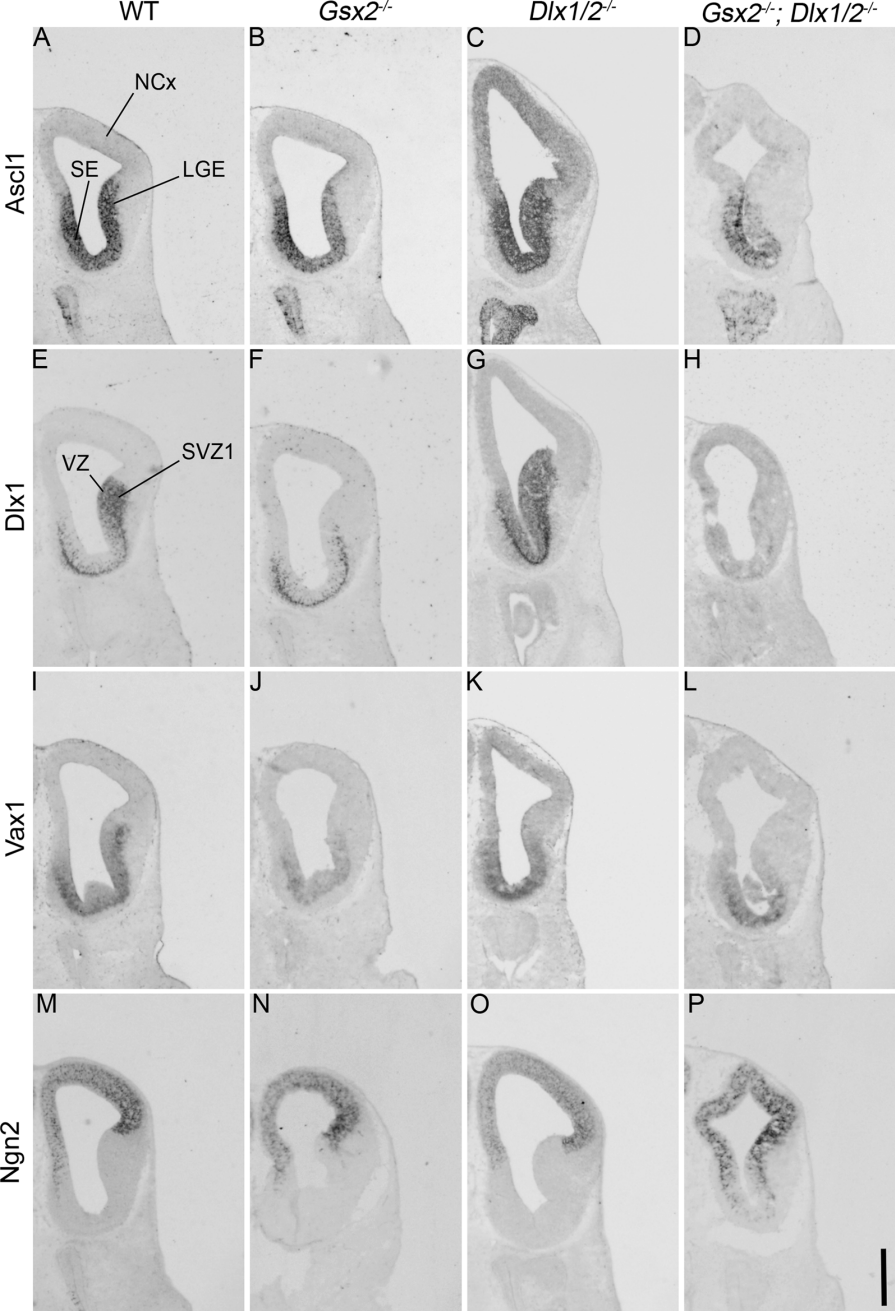
**A–P**: Combined functions of Gsx2 and Dlx1/2 define regional identity of LGE progenitor cells. In situ hybridization analysis of Ascl1, Dlx1, Vax1, and Ngn2 expression at E12.5 in the rostral telencephalon, highlighting the septum and LGE, in wild-type (WT), Gsx2^−/−^, Dlx1/2^−/−^, and Gsx2^−/−^;Dlx1/2^−/−^. Note that the LGE and septum of the mutant have lost subpallial properties and show expression of Ngn2 (pallial marker). See Figure 3 for E15.5 data. Hemisections of the telencephalon are shown. LGE, lateral ganglionic eminence; NCx, neocortex; SE, septum; SVZ1, subventricular zone 1; VZ, ventricular zone. Scale bar = 500 μm.

At E12.5, Gsx2 mutants lose expression of VZ progenitor regulators (Ascl1, Dlx1, and Vax1) in the dLGE and likewise have reduced vLGE expression of these transcription factors (Fig. 2B,F,J). Loss of Dlx1/2 has modest effects on the LGE VZ properties at E12.5 (Fig. 2C,G,K,O), but combined loss of Dlx1/2 and Gsx2 function (Gsx2;Dlx1/2 compound mutants) greatly reduced LGE VZ properties, judged from the accentuated reduction of Ascl1, Dlx1, and Vax1 expression as well as the more ventral expansion of Ngn2 (cortical) expression (Fig. 2D,H,L,P). SVZ properties of the LGE are also greatly reduced in the triple mutant, judged from expression of Arx, Dlx1, Gad1, and Vax1 (Figs. 2, 5). The LGE generates the striatum, so we assessed expression of LGE MZ markers in the Gsx2;Dlx1/2 compound mutants. Expression of Gad1 was not detected, but expression of Ebf1, FoxP4, and Islet1 were preserved, albeit reduced (see Fig. 5H,L,P,T).

**Figure 3:**
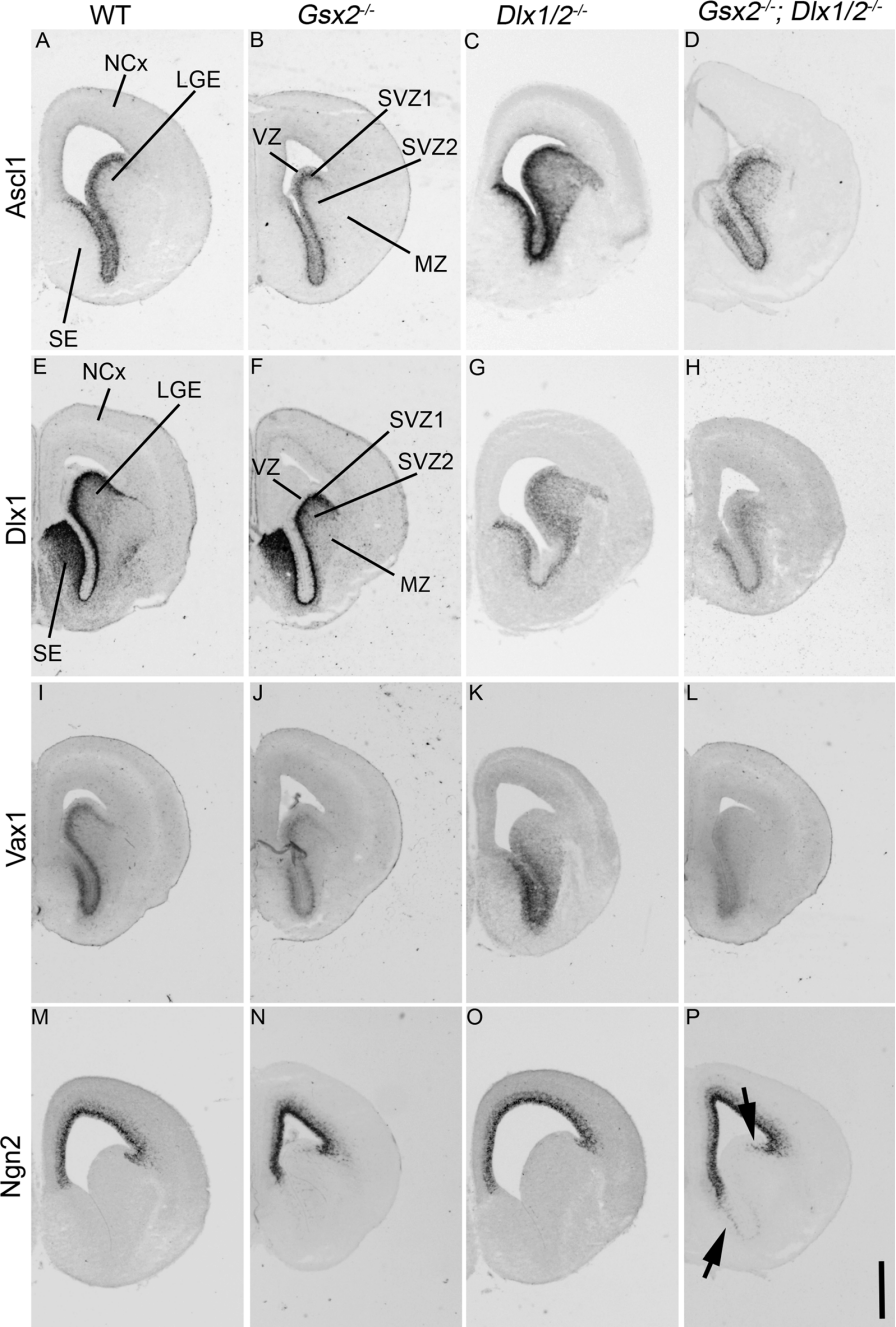
**A–P**: Combined functions of Gsx2 and Dlx1/2 define regional identity of LGE progenitor cells. In situ hybridization analysis of Ascl1, Dlx1, Vax1, Ngn2 expression at E15.5 in the rostral telencephalon, highlighting the septum and LGE, in wildp-type (WT), Gsx2^−/−^, Dlx1/2^−/−^, and Gsx2^−/−^;Dlx1/2^−/−^. Note that by this age there is nearly full recovery of the wild-type phenotype in Gsx2^−/−^, whereas in Gsx2^−/−^;Dlx1/2^−/−^ there remains ectopic Ngn2 expression in the septum and the dorsal LGE (arrows in P), as reduced expression of Vax1 and Dlx1 in the dLGE. Hemisections of the telencephalon are shown. LGE, lateral ganglionic eminence; NCx, neocortex; MZ, mantle zone; SE, septum; SVZ1, subventricular zone 1; VZ, ventricular zone. Scale bar = 1 mm.

**Figure 4:**
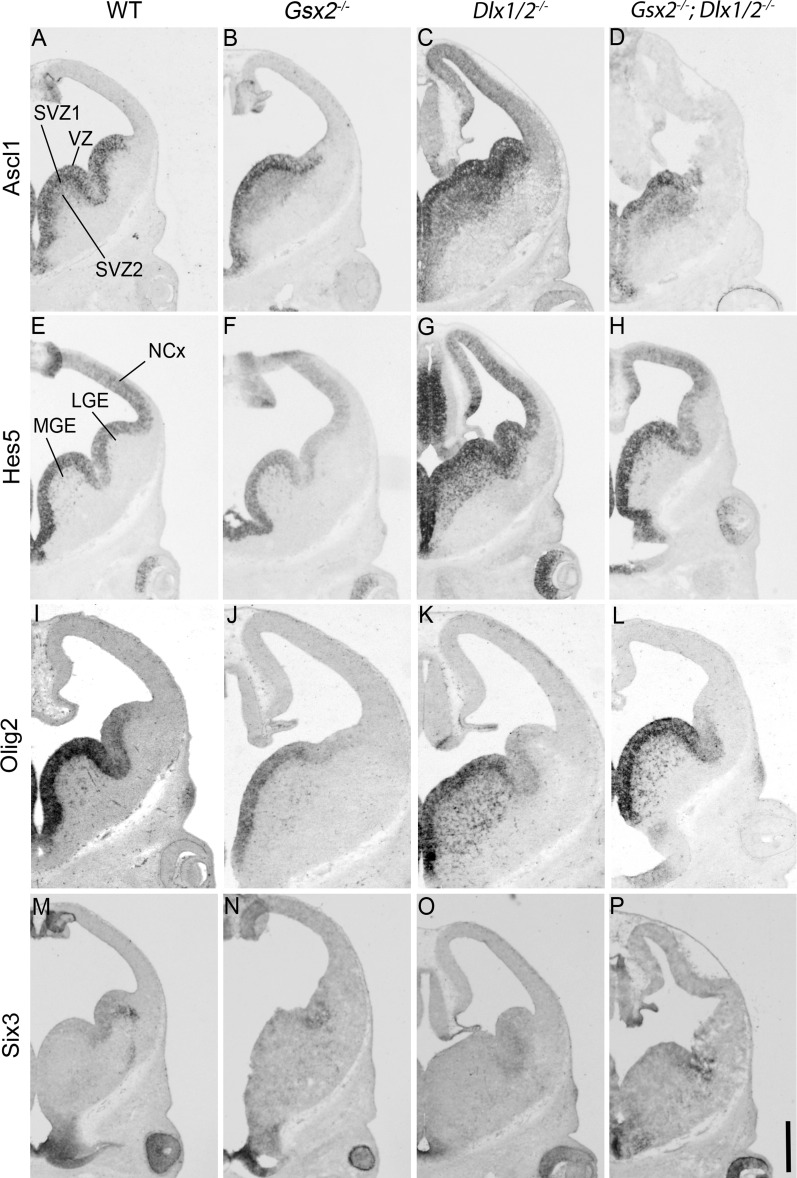
**A–P**: Gsx2 promotes and Dlx1/2 represses the Notch signaling pathway and oligodendrocyte progenitors in subpallial SVZ cells. In situ hybridization analysis of Ascl1, Hes5, Olig2, and Six3 expression at E12.5 in the middle telencephalon, highlighting the LGE, along with MGE in wild-type (WT), Gsx2^−/−^, Dlx1/2^−/−^, and Gsx2^−/−^;Dlx1/2^−/−^. Note that Ascl1, Hes5, and Olig2 MGE expression in the Gsx2^−/−^;Dlx1/2^−/−^ is restored toward WT levels (compare with the phenotype of Dlx1/2^−/−^). See Figure 12 for E15.5 data. Hemisections of the telencephalon are shown. LGE, lateral ganglionic eminence; MGE; medial ganglionic eminence; NCx, neocortex; SE, septum; SVZ1 and SVZ2, subventricular zones 1 and 2; VZ, ventricular zone. Scale bar = 500 μm.

**Figure 5:**
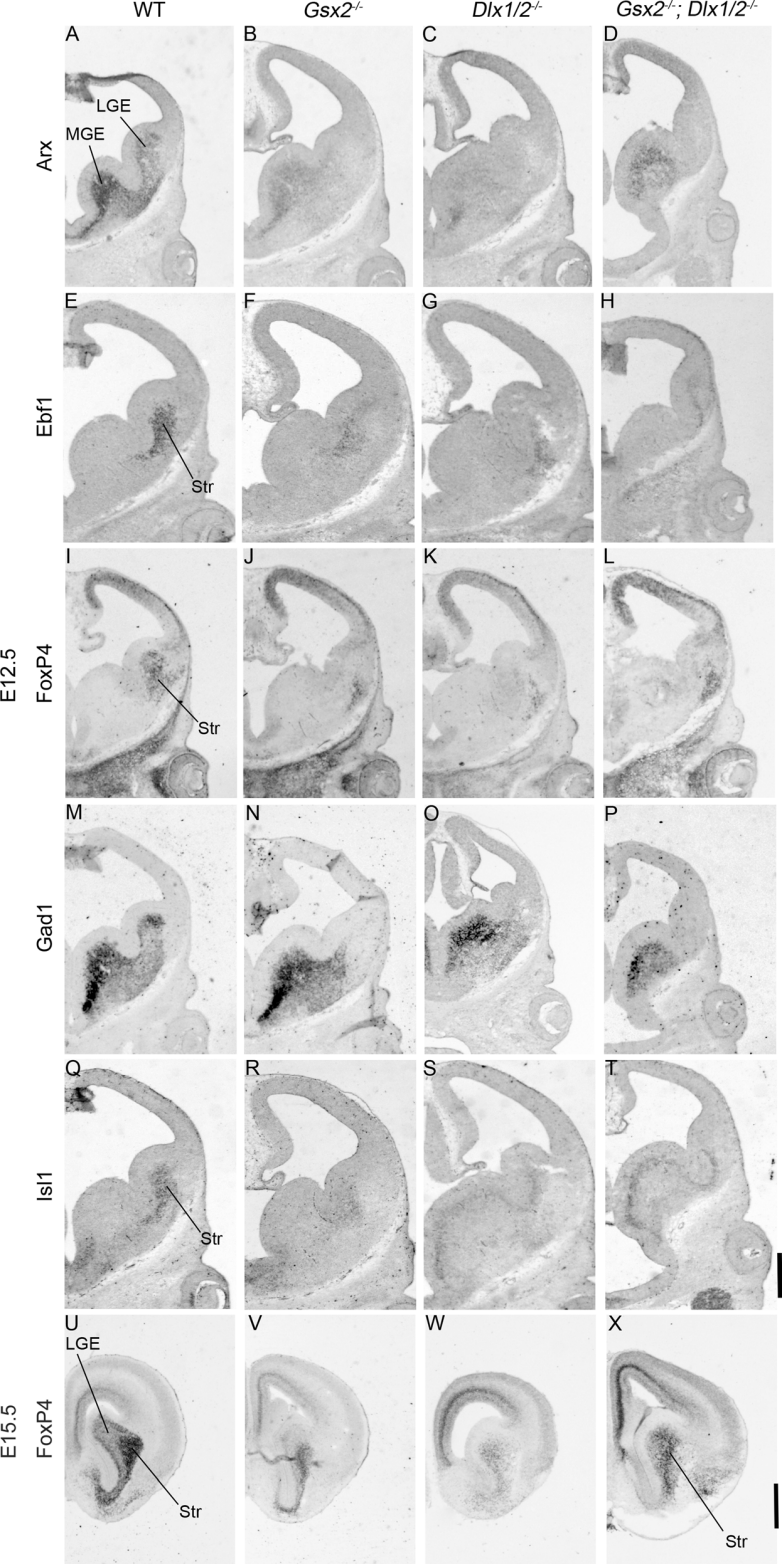
**A–X**: Gsx2 and Dlx1/2 have central roles in driving expression of Arx, Ebf1, Gad1, and Isl1 in striatal progenitors and neurons. In situ hybridization analysis of Arx, Ebf1, Foxp4, Gad1, and Isl1 expression at E12.5 (and FoxP4 at E15.5) in the middle telencephalon, highlighting the LGE (striatum) and MGE in wild-type (WT), Gsx2^−/−^, Dlx1/2^−/−^, and Gsx2^−/−^;Dlx1/2^−/−^. Note that LGE/striatal expression of Arx, Ebf1, Gad1, and Isl1 is greatly decreased in all of the mutants, whereas striatal FoxP4 expression is relatively well preserved at E12.5 and E15.5. See Figure 12 for E15.5 data. Hemisections of the telencephalon are shown. LGE, lateral ganglionic eminence; MGE, medial ganglionic eminence; NCx, neocortex; Str; striatum. Scale bars = 500 μm in T (applies to A–T); 1 mm in X (applies to U–X).

On the other hand, by E15.5, the LGE of Gsx2;Dlx1/2 compound mutant largely recovered its morphology, progenitor cell properties, and expression of some striatal markers (e.g., FoxP4; Figs. 5, 12), presumably because of the Gsx1-mediated rescue (see Toresson and Campbell, 2001; Yun et al., 2003). However, despite the recovery of some LGE/striatal properties, expressions of Arx and Gad1 were less than in the single mutants (Figs. 5D,P, 12D,L); thus, together, Gsx2 and Dlx1/2 have a central role in promoting expression of Arx and Gad1 in striatal progenitors and neurons.

**Figure 6:**
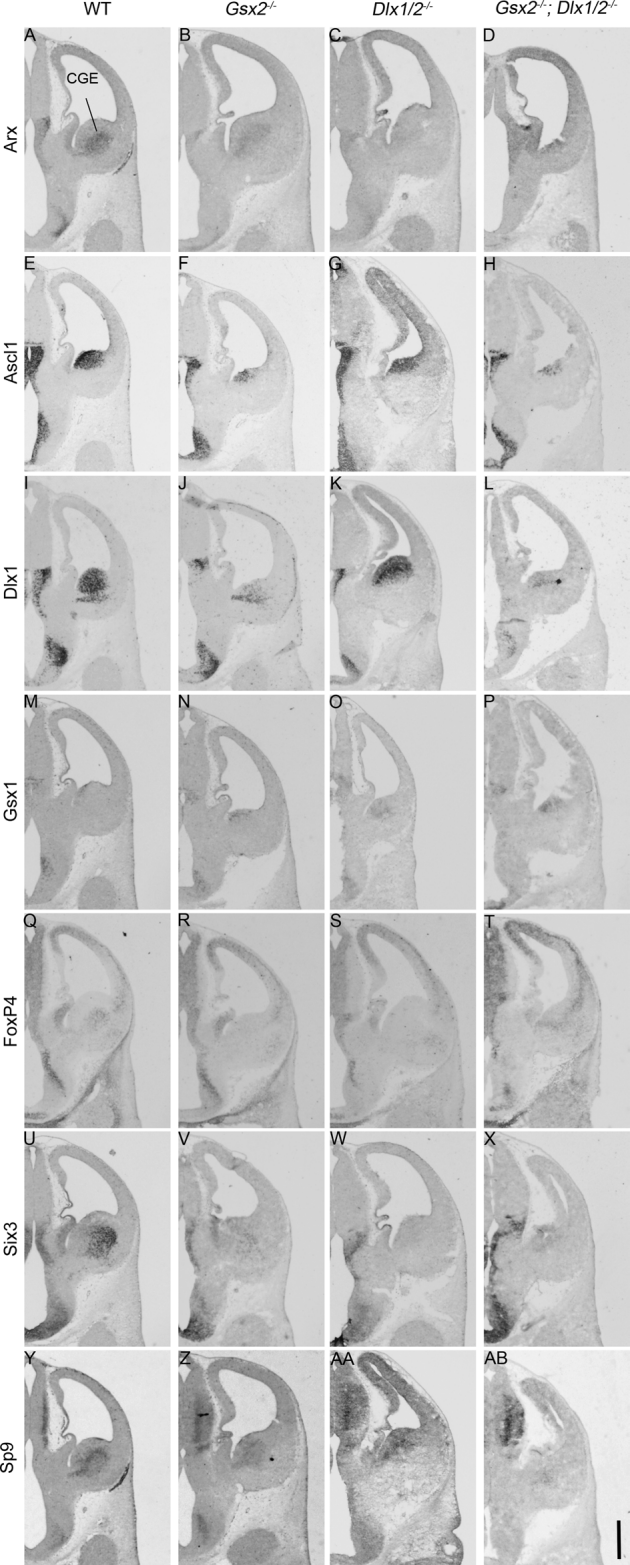
**A–AB**: Combined functions of Gsx2 and Dlx1/2 define regional identity of CGE progenitor cells. In situ hybridization analysis of Arx, Ascl1, Dlx1, Gsx1, FoxP4, Six3, and Sp9 expression at E12.5 in the caudal telencephalon, highlighting the CGE in wild-type (WT), Gsx2^−/−^, Dlx1/2^−/−^, and Gsx2^−/−^;Dlx1/2^−/−^. Note that the CGE of the Gsx2^−/−^;Dlx1/2^−/−^ mutant has lost most of its subpallial properties. See Figure 10 for E15.5 data. Hemisections of the telencephalon are shown. CGE, caudal ganglionic eminence. Scale bar = 500 μm.

**Figure 7:**
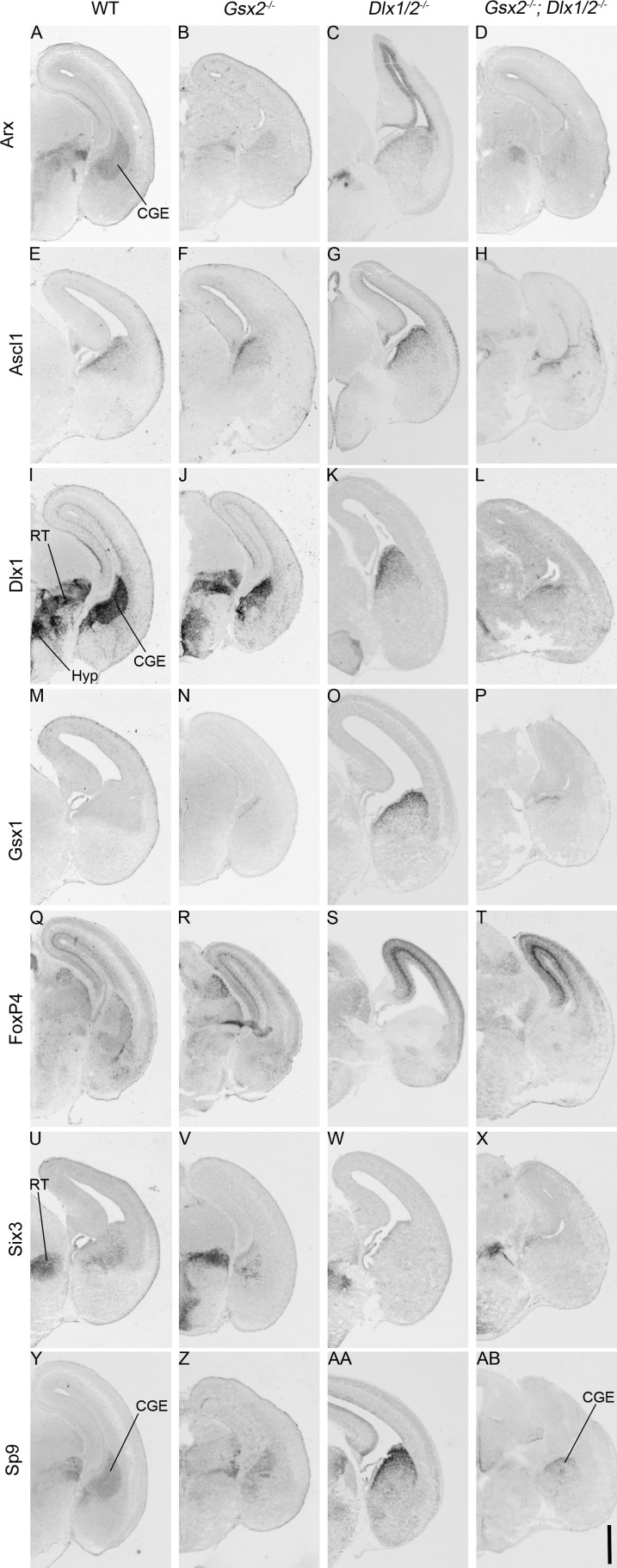
**A–AB**: Combined functions of Gsx2 and Dlx1/2 define regional identity and differentiation of CGE progenitor cells. In situ hybridization analysis of Arx, Ascl1, Dlx1, Gsx1, FoxP4, Six3, and Sp9 expression at E15.5 in the caudal telencephalon, highlighting the CGE in wild-type (WT), Gsx2^−/−^, Dlx1/2^−/−^, and Gsx2^−/−^; Dlx1/2^−/−^. Note that the CGE of the Gsx2^−/−^;Dlx1/2^−/−^ mutant has lost most of its subpallial properties, except for residual Ascl1, Dlx1, Gsx1, and Sp9 expression in progenitor cells. Hemisections of the telencephalon are shown. CGE, caudal ganglionic eminence; Hyp, hypothalamus; RT, reticular thalamus. Scale bar = 1 mm.

The CGE is largely a caudal extension of the LGE, although the neuronal output of these two progenitor zones differs (their principal derivatives include: LGE, striatum; CGE, cortical interneurons; Flames et al., 2007; Waclaw et al., 2009; Fishell and Rudy, 2011). Given the severe LGE defects in the Gsx2;Dlx1/2 compound mutants, we examined the CGE phenotype at E12.5 and E15.5 (Figs. 6, 7). Previous studies demonstrated CGE deficiencies in Gsx2 and Dlx1/2 mutants (Long et al., 2009b; Xu et al., 2010), whose nature was confirmed here by the reduced CGE expression of Arx, Ascl1, Dlx1, Six3, and Sp9 (Figs. 6, 7). The CGE in the Gsx2;Dlx1/2 compound mutants was severely hypoplastic based on morphology; on loss of Arx, Six3, and Sp9 expression; and on extension of FoxP4^+^ pallial expression into the region (Figs. 6D,X,AB, 7D,X,AB). A CGE rudiment may persist based on residual Ascl1, Dlx1, and Gsx1 expression, although this could be from the caudal-most MGE (Figs. 6H,L,P, 7H,L,P). At E15.5, unlike the case in the LGE, we did not detect a recovery of CGE properties in the Gsx2;Dlx1/2 compound mutants (there was a modest recovery in the Gsx2 mutant; Fig. 7).

Overall, at E12.5 the Gsx2;Dlx1/2 compound mutants had a severe defect in regional specification of the LGE and CGE that was more severe than that in either the Dlx1/2 or the Gsx2 mutants. By E15.5, although there was partial recovery of the LGE phenotype, the CGE phenotype was not rescued. Furthermore, together, Gsx2 and Dlx1/2 were essential for expression of Gad1 in LGE- and CGE-derived neurons.

### Gsx2 promotes and Dlx1/2 represses the Notch signaling pathway and oligodendrocyte progenitors in subpallial SVZ cells

Previously we showed that Dlx1/2 mutants have elevated levels of Notch signaling in their SVZ, based on increased expression of Ascl1, Delta1, and Hes5 (Yun et al., 2002; Long et al., 2009a,b). The Gsx2 mutant had reduced expression of Ascl1 and Hes5 (Fig. 4B,F). Given that Dlx1/2 mutants have elevated Gsx2 expression (Yun et al., 2002; Long et al., 2009a), we tested whether Gsx2;Dlx1/2 compound mutants have normalized Notch signaling. To do this, we studied the MGE at E12.5, because the LGE and CGE in the triple mutant are nearly eliminated. Indeed, Ascl1 and Hes5 expression was restored toward WT levels (Fig. 4D,H). In addition, we previously showed that Dlx1/2 mutants have elevated levels of oligodendrogensis in their MGE because they overexpress Olig2 (Petryniak et al., 2007; Fig. 4K). On the contrary, here we found that Gsx2 mutants had reduced Olig2 expression (Fig. 4J). Gsx2;Dlx1/2 compound mutants have normalized level of Olig2 expression (Fig. 4L).

Therefore, major features of subpallial progenitors were disrupted in Gsx2;Dlx1/2 compound mutants (Arx, Gad1, Gsx1, Pbx1, Otp, Sp8, Sp9, Vax1 expression). However, some fundamental features (Ascl1 and Olig2 expression) were preserved, showing that part of the subpallial progenitor transcriptional program remains. Furthermore, removing Gsx2 function in the Dlx1/2 mutants rescued the elevated levels of Olig2 and Hes5 expression (Notch signaling). Next, we investigated whether removing Gsx2 function in the Dlx1/2 mutants rescued abnormal expression of Gsx1, Gbx1, and Otp.

### Role of Gsx2 in Dlx1/2-mediated repression of Gsx1, Gbx1, and Otp expression in subpallial progenitors

**Figure 8:**
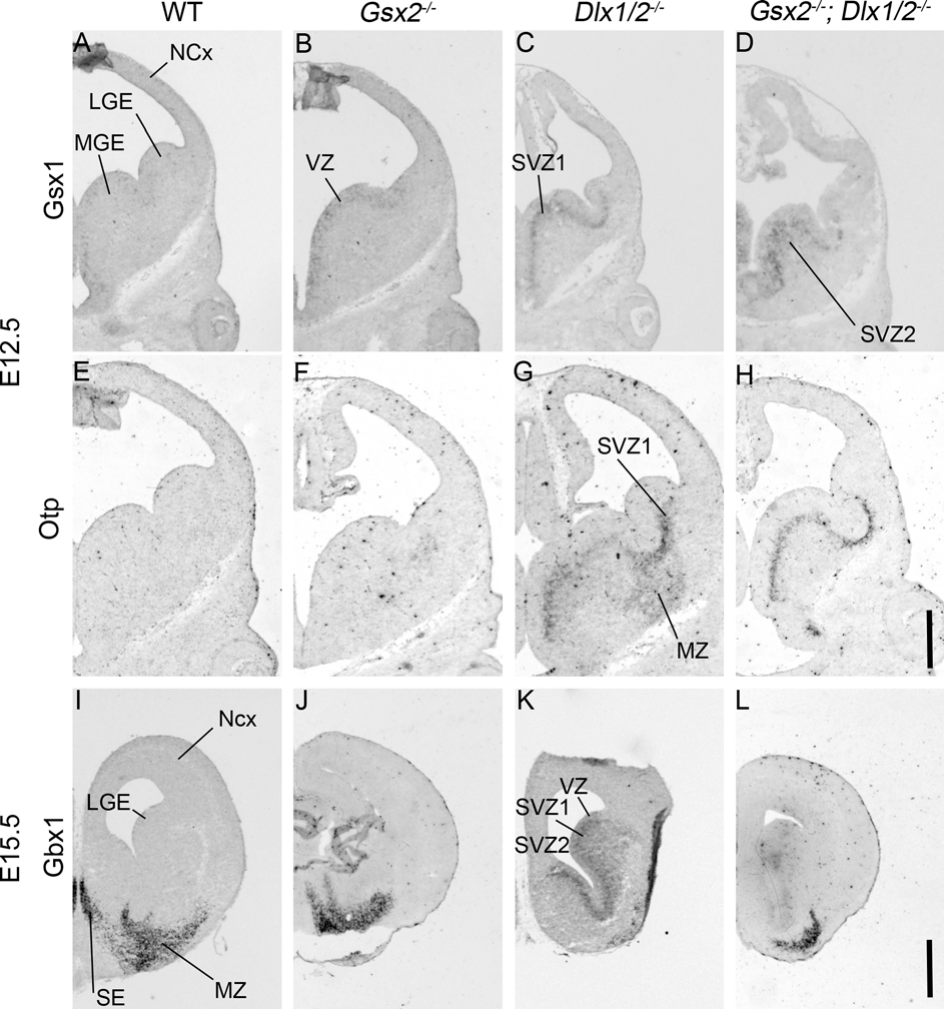
**A–L**: Gsx2 is required for Dlx1/2-mediated repression of Gbx1, but not of Gsx1 and Otp expression, in subpallial progenitors. In situ hybridization analysis of Gsx1 and Otp expression at E12.5 and Gbx1 at E15.5, in the middle telencephalon, highlighting the LGE and MGE in wild-type (WT), Gsx2^−/−^, Dlx1/2^−/−^, and Gsx2^−/−^;Dlx1/2^−/−^. Abnormal expression of Gbx1 in the Dlx1/2^−/−^ LGE is reversed in Gsx2^−/−^;Dlx1/2^−/−^, whereas the Gsx1 and Otp phenotypes are not reversed. See Figure 7 for E15.5 Gsx1 data. Hemisections of the telencephalon are shown. LGE, lateral ganglionic eminence; MGE, medial ganglionic eminence; MZ, mantle zone; NCx, neocortex; SE, septum; SVZ1 and SVZ2, subventricular zones 1 and 2; VZ, ventricular zone. Scale bars = 500 μm in H (applies to A–H); 1 mm in L (applies to I–L).

Dlx1/2 mutants overexpressed Gsx1, and ectopically expressed Gbx1 and Otp, in subpallial progenitors (Fig. 1H; Long et al., 2009a). To assess whether Gsx2 overexpression in the Dlx1/2 mutant mediated the abnormal Gsx1, Gbx1, and Otp expression, we studied the Gsx2;Dlx1/2 compound mutants. Gsx1 overexpression and Otp ectopic expression were not rescued in the triple mutant (Fig. 8D,H). On the other hand, Gbx1 ectopic expression was rescued (Fig. 8L). Thus, ectopic expression of Gbx1, like overexpression of Ascl1, Hes5, and Olig2 (Fig. 4), was rescued by removing Gsx2 from the Dlx1/2 mutant.

### Combined functions of Gsx2 and Dlx1/2 are required to maintain Arx and Gad1 expression in the MGE

**Figure 9:**
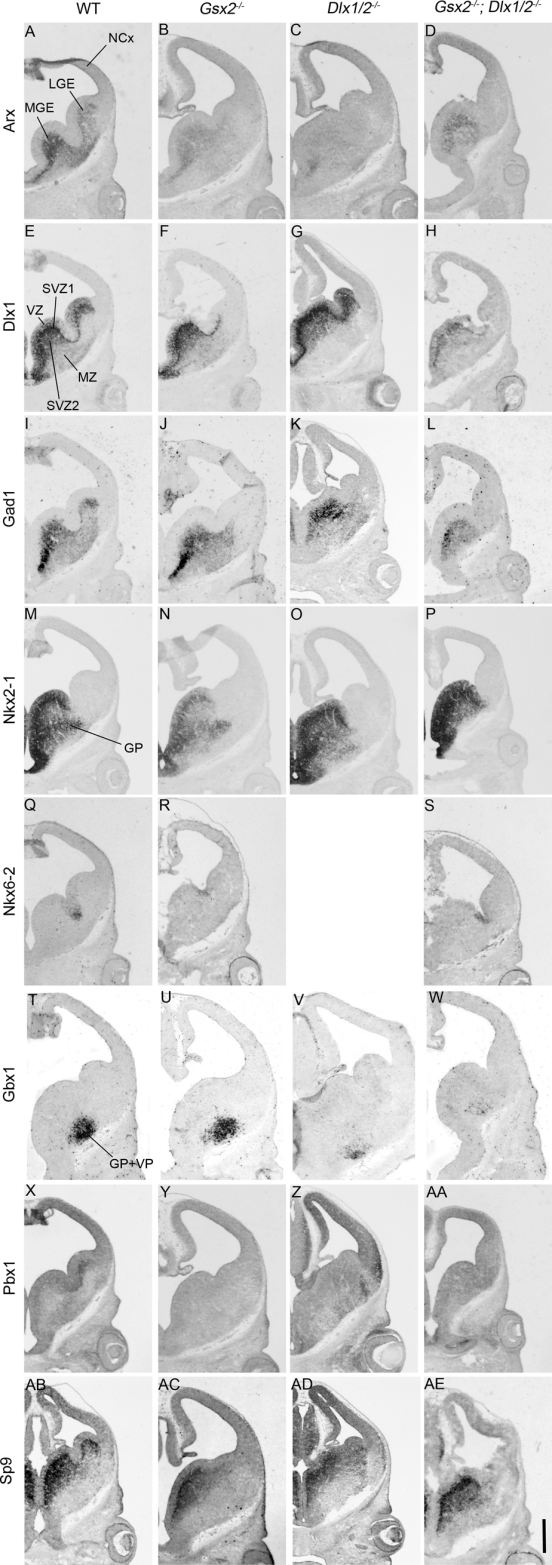
**A–AC**: Relatively mild MGE phenotypes in the Gsx2^−/−^, Dlx1/2^−/−^, and Gsx2^−/−^;Dlx1/2^−/−^ mutants. In situ hybridization analysis of Arx, Dlx1, Gad1, Nkx2-1, Nkx6-2, Gbx1, FoxP4, Pbx1, and Sp9 expression at E12.5 in the middle telencephalon, highlighting the LGE and MGE in wild-type (WT), Gsx2^−/−^, Dlx1/2^−/−^, and Gsx2^−/−^;Dlx1/2^−/−^. Although MGE differentiation is abnormal in the Dlx1/2 mutant (e.g., small globus pallidus, reduced Arx and Gad1 expression), the phenotype is not strongly altered by removing Gsx2. See Figure 10 for E15.5 data. Hemisections of the telencephalon are shown. LGE, lateral ganglionic eminence; MGE, medial ganglionic eminence; MZ, mantle zone; NCx, neocortex; GP, globus pallidus; SVZ1 and SVZ2, subventricular zones 1 and 2; VP, ventral pallidum; VZ, ventricular zone. Scale bar = 500 μm.

Specification of MGE progenitors (based on expression of Nkx2.1 and Lhx6), unlike the LGE and CGE, was not strongly affected by loss of either Gsx2 or Dlx1/2 (Yun et al., 2003; Long et al., 2009b). To test whether this could be due to compensation by Gsx2 and Dlx1/2, we studied the Gsx2;Dlx1/2 compound mutants. However, as in the individual mutants, the triple mutant continued to show relative normal indices of MGE regional identify, including VZ expression of Nkx2.1, Nkx6.2, and Olig2 (Figs. 4L, 9P,S).

**Figure 10:**
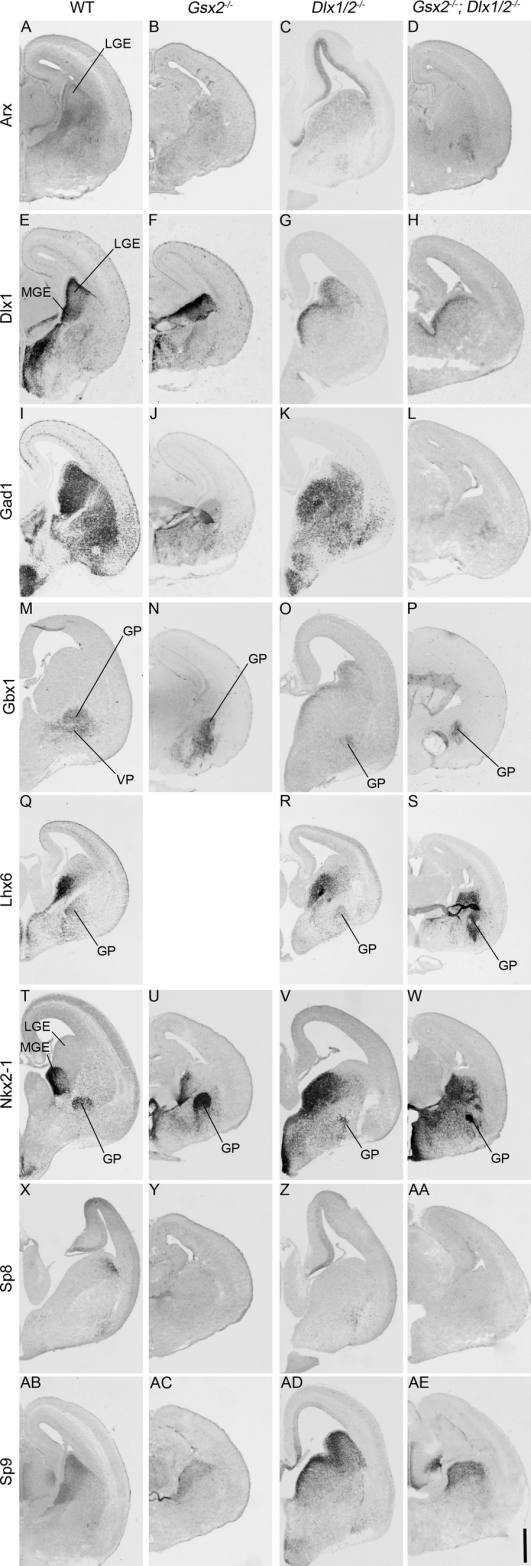
**A–AE**: Relatively mild MGE phenotypes in the Gsx2^−/−^, Dlx1/2^−/−^, and Gsx2^−/−^;Dlx1/2^−/−^ mutants. In situ hybridization analysis of Arx, Dlx1, Gad1, Lhx6, Nkx2-1, Nkx6-2, Gbx1, FoxP4, Pbx1, and Sp9 expression at E15.5 in the middle telencephalon, highlighting the LGE and MGE in wild-type (WT), Gsx2^−/−^, Dlx1/2^−/−^, and Gsx2^−/−^;Dlx1/2^−/−^. Although MGE differentiation is abnormal in the Dlx1/2 mutant (e.g., small globus pallidus, reduced Arx and Gad1 expression), the phenotype is not strongly altered by removing Gsx2. Expression of Lhx6 in the Gsx2^−/−^ is not shown; previously, we found it to be normal at E11.5 and E18.5 (Yun et al., 2003). Hemisections of the telencephalon are shown. LGE, lateral ganglionic eminence; MGE, medial ganglionic eminence; GP, globus pallidus; VP, ventral pallidum. Scale bar = 1 mm.

In the Dlx1/2 mutant, although MGE VZ specification did not appear to be altered, MGE SVZ properties were altered, including elevated Ascl1, Hes5 and Olig2 expression (described above; Fig. 4C,G,K) and reduced Arx and Gad1 expression (Cobos et al., 2005b; Long et al., 2009b). At E12.5, the Gsx2;Dlx1/2 compound mutant MGE SVZ had “normalized” Ascl1, Hes5, and Olig2 expression and continued to express Arx and Gad1 (Figs. 4D,H,L, 9D,L). However, by E15.5, Arx and Gad1 expression in the Gsx2;Dlx1/2 compound mutant was reduced compared with that in either the Gsx2 or the Dlx1/2 mutant (Fig. 10D,L), although other MGE SVZ properties were maintained (Ascl1, Hes5, Lhx6, Nkx2.1 Olig2, and Sp9; Fig. 10S,W,AE, and not shown).

MGE MZ properties were also defective in Dlx1/2 mutants, including a small globus pallidus (Gbx1^+^, Lhx6^+^, Nkx2.1^+^; Long et al., 2009b; Fig. 10O,R,V). This defect was similar in the Gsx2;Dlx1/2 compound mutant, although there may be increased numbers of Lhx6^+^ and Nkx2.1^+^ cells in the MZ at E15.5 (Fig. 10S,W). Tangential migration of Lhx6^+^ cells to the cortex remained strongly blocked (Fig. 10S); this should be compared with a partial rescue of this phenotype in the Gsx1;Dlx1/2 compound mutant (discussed below; see Fig. 14).

### Transcription factor expression in septal progenitor and mantle cells at E12.5 and E15.5 in Gsx2, Dlx1/2, and Gsx2;Dlx1/2 mutants

**Figure 11:**
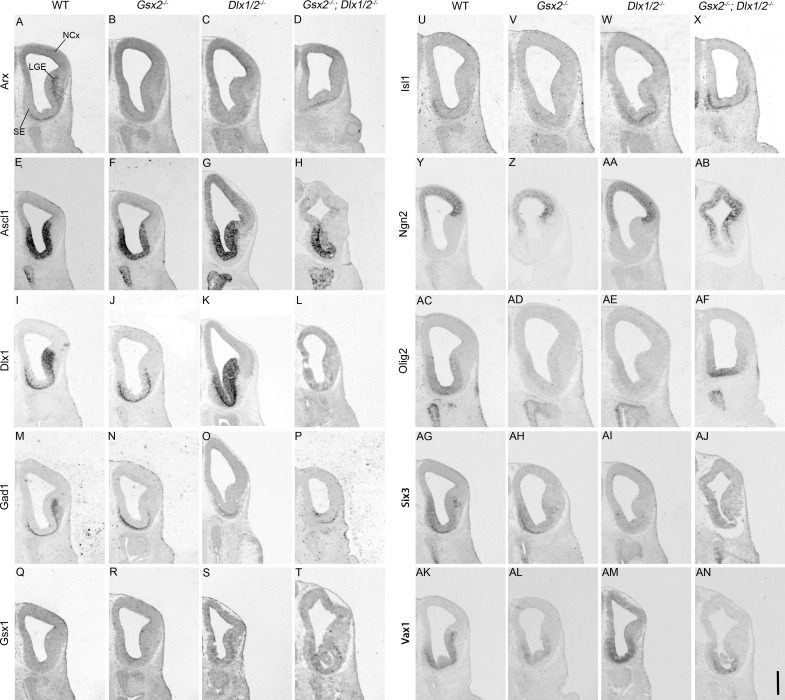
**A–AN**: Dlx1/2 and Gsx2 are critical for septal development. In situ hybridization analysis of Arx, Ascl1, Dlx1, Gad1, Gsx1, Isl1, Ngn2, Olig2, Six3, and Vax1 at E12.5 in the rostral telencephalon, highlighting the LGE and septum in wild-type (WT), Gsx2^−/−^, Dlx1/2^−/−^, and Gsx2^−/−^;Dlx1/2^−/−^. Each single mutant shows gene expression defects, which are amplified in Gsx2^−/−^;Dlx1/2^−/−^. See Figure 12 for E15.5 data. Hemisections of the telencephalon are shown. LGE, lateral ganglionic eminence; NCx, neocortex; SE, septum. Scale bar = 500 μm.

Gsx2 function in septal development has previously not been reported. At E12.5, Gsx2 mutants showed reduced expression of Arx, Hes5, Islet1, Olig2, and Vax1, whereas Ascl1, Dlx1, Foxp4, GAD1, Ngn2, and Six3 expression did not appear modified (Figs. 2, 11; not shown). Pbx1 and Sp9 septal expression was just beginning, so it was difficult to discern a phenotype (not shown).

**Figure 12:**
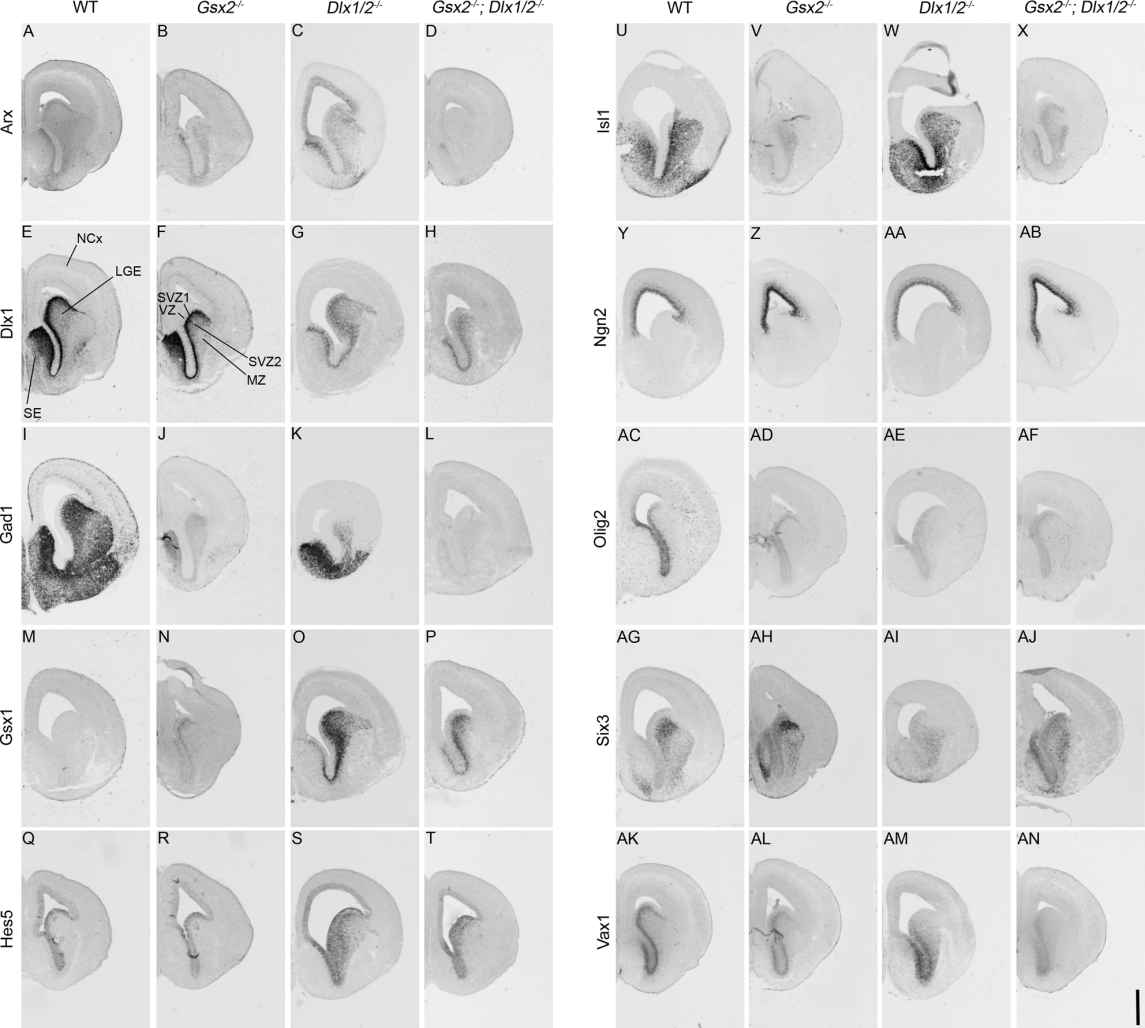
**A–AN**: Gsx2 and Dlx1/2 have central roles in maintaining expression of Gad1 and other features of septal and striatal development. In situ hybridization analysis of Arx, Dlx1, Gad1, Gsx1, Isl1, Ngn2, Olig2, Six3, and Vax1 expression at E15.5 in the rostral telencephalon, highlighting the LGE (striatum) and septum in wild-type (WT), Gsx2^−/−^, Dlx1/2^−/−^, and Gsx2^−/−^;Dlx1/2^−/−^. Note the loss of Arx, Gad1, and Isl1 expression in the LGE/striatum and septum. Hemisections of the telencephalon are shown. LGE, lateral ganglionic eminence; MGE, medial ganglionic eminence; NCx, neocortex; Str, striatum. Scale bar = 1 mm.

By E15.5 in the Gsx2 mutant, Arx expression appeared to be restored (as in the LGE), and Gsx1 was increased (Fig. 12B,N). However, expression was reduced for Ascl1 (thinner VZ), FoxP4, Islet1, Olig2 (ventral VZ), Sp8 (SVZ and MZ), Sp9 (MZ), and Vax1 (Figs. 3, 12, and not shown). Expression appeared normal for Dlx1, GAD1, Gbx1, Ngn2, and Pbx1 (Fig. 12, and not shown). Expression of Six3 may be increased (Fig. 12AH).

Dlx1/2 mutants have septal defects (Long et al., 2009a); here we extended these observations at E12.5 and E15.5. At E12.5, there was reduced expression of Arx, Olig2, and Six3 (Fig. 11C,AE,AI); increased expression of Ascl1, Gsx2, Islet1, and Sp9 (Fig. 11G,S,W, and not shown); no obvious change in expression of Dlx1, FoxP4, GAD1, Pbx1, and Vax1 (Fig. 11K,O,AM, and not shown). At E15.5, there was reduced expression of Dlx1, Gbx1 (MZ) Olig2 (slight), Pbx1, and Six3 (Fig. 12G,AE,AI, and not shown); increased expression of Ascl1, Gsx1, Gsx2, Islet1, and Sp9 (Fig. 12O,W, and not shown); no obvious change in expression of GAD1, Arx, FoxP4, and Vax1 (Fig. 12C,K,AM, and not shown); and ectopic expression of Otp (not shown).

The Gsx2;Dlx1/2 compound mutant septum showed complex and time-dependent phenotypes. At E12.5, the phenotype was more severe for Ascl1 (reduced), Dlx1 (reduced), GAD1 (reduced), Gsx1 (increased), Ngn2 (increased), and Vax1 (reduced; Figs. 11H,L,P,T,AB,AN, 16). For other genes, the compound mutant phenocopied the Dlx1/2 mutant: Arx (decreased), Islet1 (increased), Six3 (decreased), Sp9 (increased); or it phenocopied the Gsx2 mutant: Arx (decreased), Ngn2 (ectopic, but more severe), Vax1 (decreased; Fig. 11D,X,AB,AJ,AN, and not shown). There may be partial rescue of the Olig2 expression in the compound mutant (Fig. 11AF).

At E15.5, the Gsx2;Dlx1/2 compound mutant's septum showed a more severe reduction of GAD1, Arx, FoxP4, Pbx1, Sp8, Sp9, and Vax1 (Fig. 12D,L,AN, and not shown). For other genes, the compound mutant phenocopied the Dlx1/2 mutant: Dlx1 (reduced), Gbx1 (reduced), Gsx1 (increased), Islet1 (increased), Otp (increased), Sp9 (increased; Fig. 12H,P,X, and not shown); or phenocopied the Gsx2 mutant: Six3 (increased), Vax1 (reduced; Fig. 12AJ,AN). There was an intermediate phenotype for Islet1: increased in the SVZ (like Dlx1/2) and decreased in the MZ (like Gsx2). Ngn2 was not ectopically expressed (Fig. 12AB). Ascl1 and Hes5 expression appeared intermediate between the Gsx2 mutant and the Dlx1/2 mutant phenotypes (Fig. 12T, and not shown). Nkx2.1 (medial septum MZ) and Olig2 (VZ) expression was not clearly changed in the compound mutant (Fig. 12AF, and not shown).

### Analysis of Gsx1, Dlx1/2, and Gsx1;Dlx1/2 mutants: partial rescue of the MGE and tangential migration of Lhx6^+^ cells to the cortex

Previous studies have failed to identify strong molecular or cellular defects in Gsx1 mutant basal ganglia, aside from ectopic Dbx1 expression (Toresson and Campbell, 2001; Yun et al., 2003). Here we investigated Gsx1 function because its expression is increased in the Dlx1/2 mutants (Long et al., 2009a,b). We studied the phenotype of the Gsx1;Dlx1/2 compound mutants to determine whether some of the Dlx1/2 phenotype was caused by overexpression of Gsx1. Analysis was performed at E15.5.

**Figure 13:**
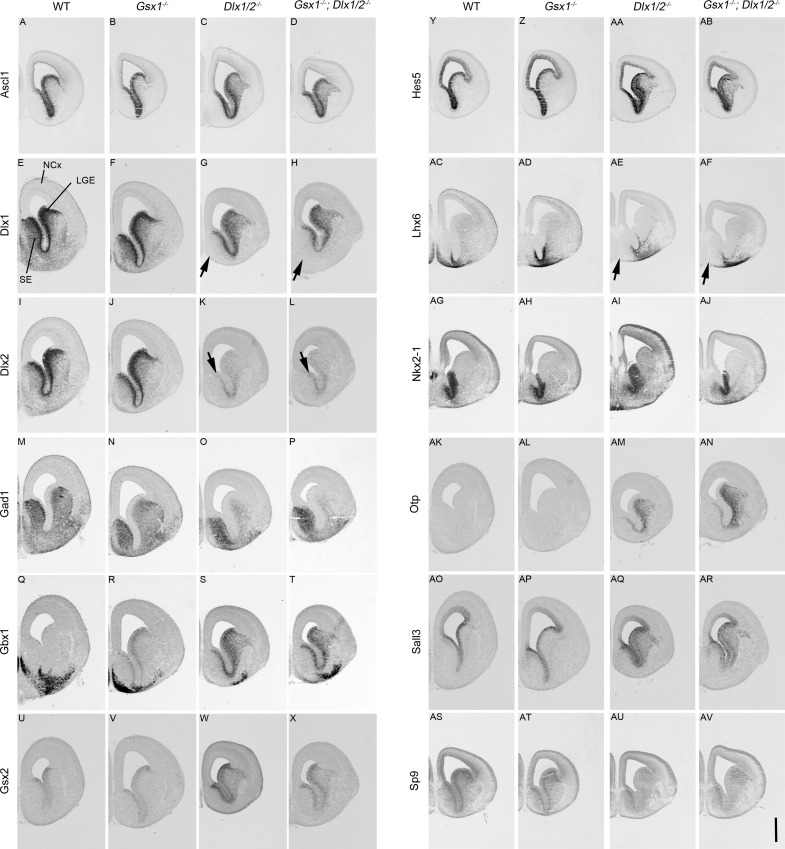
**A–AV**: Gsx1, in combination with Dlx1/2, regulates septal development. In situ hybridization analysis of Ascl1, Dlx1, Dlx2, Gad1, Gbx1, Gsx2, Hes5, Lhx6, Nkx2-1, Otp, Sall3, and Sp9 at E15.5 in the rostral telencephalon, highlighting the LGE and septum in wild-type (WT), Gsx1^−/−^, Dlx1/2^−/−^, and Gsx1^−/−^;Dlx1/2^−/−^. The Gsx1 septum had more obvious phenotypes than the LGE (see Results); arrows point to reduced Dlx1, Dlx2, and Lhx6 expression in the septum (G,H,K,L,AE,AF). Hemisections of the telencephalon are shown. LGE, lateral ganglionic eminence; NCx, neocortex; SE, septum. Scale bar = 1 mm.

First, we identified some subtle phenotypes in the Gsx1 mutant. The septum had the most obvious phenotypes, with reduced expression of Dlx1 and Dlx2 in the VZ; reduced Hes5 in the SVZ; and reduced Dlx2, Gbx1, Lhx6, and Nkx2.1 in the MZ (particularly medial septum); Gbx1 expression was increased in the SVZ (Fig. 13R). The LGE showed reduced expression of Dlx1 and Dlx2 in the VZ (ventral more severe than dorsal; Fig. 13F,J) and reduced Gad1 expression in the SVZ (Fig. 13N); Gbx1 expression was increased in the VZ (Fig. 13R).

**Figure 14:**
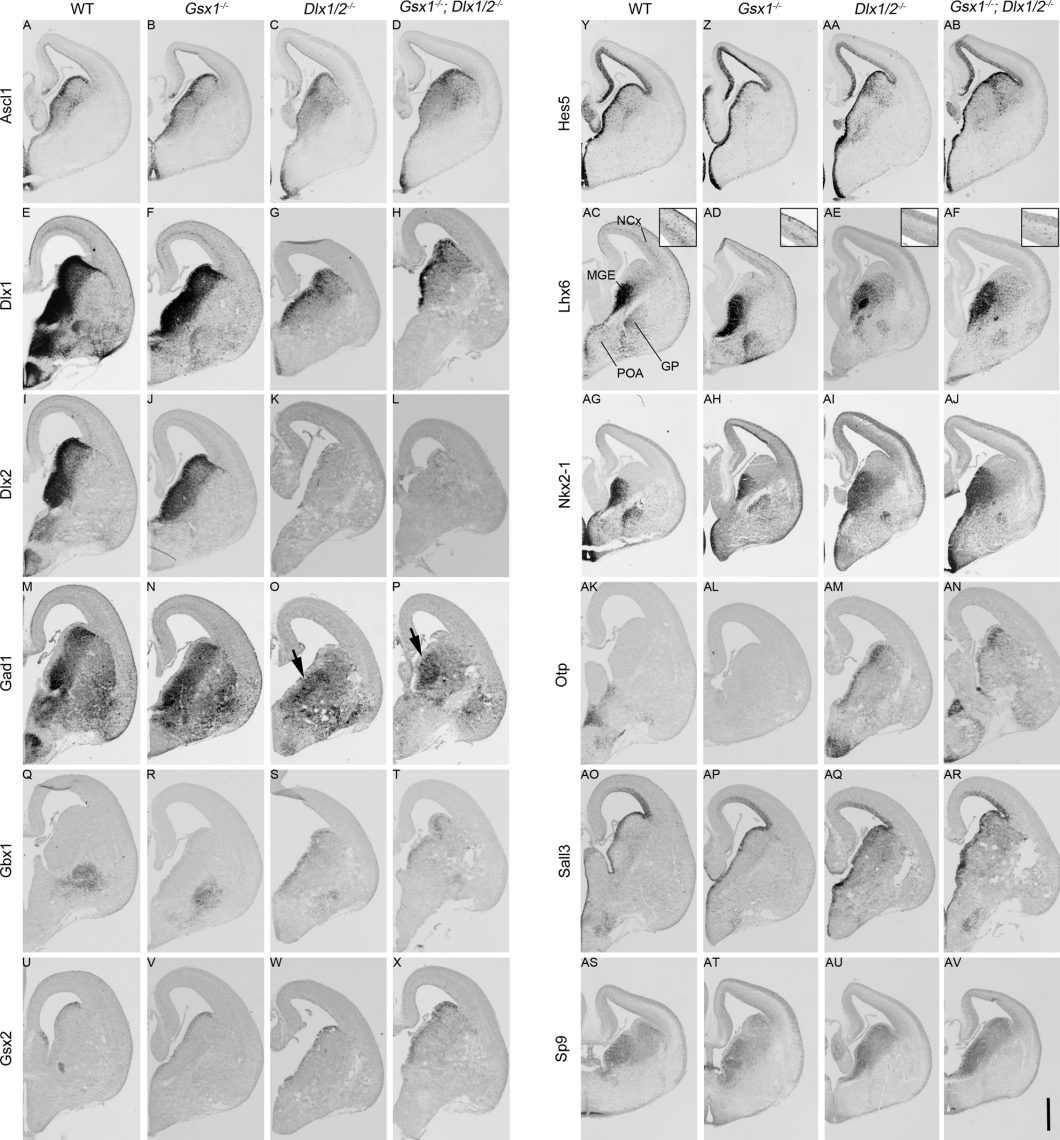
**A–AV**: Removal of Gsx1 partially rescues MGE differentiation in Dlx1/2 mutants. In situ hybridization analysis of Ascl1, Dlx1, Dlx2, Gad1, Gbx1, Gsx2, Hes5, Lhx6, Nkx2-1, Otp, Sall3, and Sp9 at E15.5 in the middle telencephalon, highlighting the LGE and MGE in wild-type (WT), Gsx1^−/−^, Dlx1/2^−/−^, and Gsx1^−/−^;Dlx1/2^−/−^. Note the partial rescue of Lhx6^+^ cells in the neocortex (higher magnifications in AC–AF) and the increased expression of Gad1 and Lhx6 in the SVZ of the MGE. Hemisections of the telencephalon are shown. GP, globus pallidus; MGE, medial ganglionic eminence; NCx, neocortex; POA, preoptic area. Scale bar = 1 mm.

Next, we assessed whether removing Gsx1 expression in the Dlx1/2 mutants rescued any of the Dlx1/2 mutant phenotypes. Remarkably, there was partial reduction of tangential migration of Lhx6^+^ cells into the cortex of Gsx1;Dlx1/2 compound mutants (Fig. 14AF). Associated with this was an increase in Lhx6 expression in the progenitor zones of the MGE to a level similar to the wild type (Fig. 14AF). Likewise, in the Gsx1;Dlx1/2 compound mutant MGE, Hes5 expression in the SVZ was reduced toward wild-type levels, and GAD1 expression was increased toward wild-type levels (Fig. 14P,AB). This provides evidence that elevated Gsx1 expression in the Dlx1/2 mutant contributes to MGE differentiation and migration defects.

**Figure 15:**
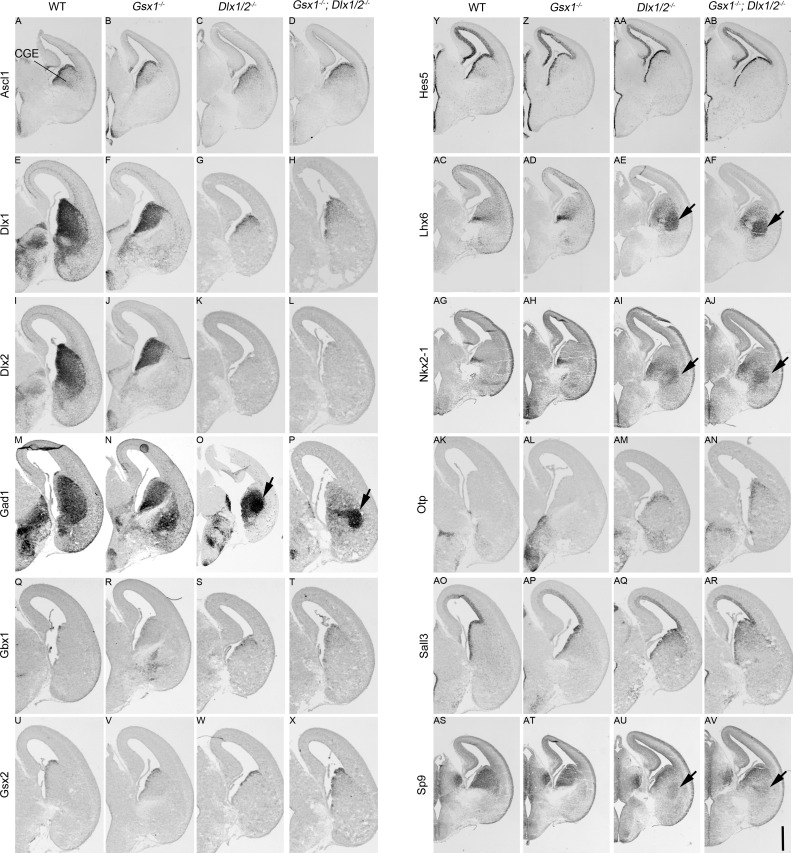
**A–AN**: Although loss of Gsx1 partially rescues the Dlx1/2^−/−^ MGE, Lhx6^+^, Nkx2-1^+^, Gad1^+^, and Sp9^+^ cells continue to migrate ectopically in the CGE (arrows in O,P,AE,AF,AI,AJ,AU,AV). In situ hybridization analysis of Ascl1, Dlx1, Dlx2, Gad1, Gbx1, Gsx2, Hes5, Lhx6, Nkx2-1, Otp, Sall3, and Sp9 at E15.5 in the caudal telencephalon, highlighting the CGE in wild-type (WT), Gsx1^−/−^, Dlx1/2^−/−^, and Gsx1^−/−^;Dlx1/2^−/−^. Hemisections of the telencephalon are shown. CGE, caudal ganglionic eminence. Scale bar = 1 mm.

In addition to the partial rescue of the MGE phenotypes, there was subtle rescue of septal and CGE phenotypes, including expression of Ascl1 (VZ of septum and CGE; Figs. 13D, 15D), Dlx1 (VZ of septum, MGE and CGE; Figs. 13H, 14H, 15H), Hes5 (SVZ of MGE; Fig. 14AB), and Sall3 (VZ of septum; Fig. 13AR). By contrast with partial rescue in the MGE, septum, and CGE, some phenotypes were worsened in the triple mutants, including expression of Ascl1 (VZ of vLGE; Fig. 13D), Hes5 (SVZ of septum; Fig. 13AB), and Sall3 (VZ of vLGE; Fig. 13AV). Finally, we did not observe any clear rescue of LGE phenotypes (Long et al., 2009a; Fig. 13).

## DISCUSSION

**Figure 16:**
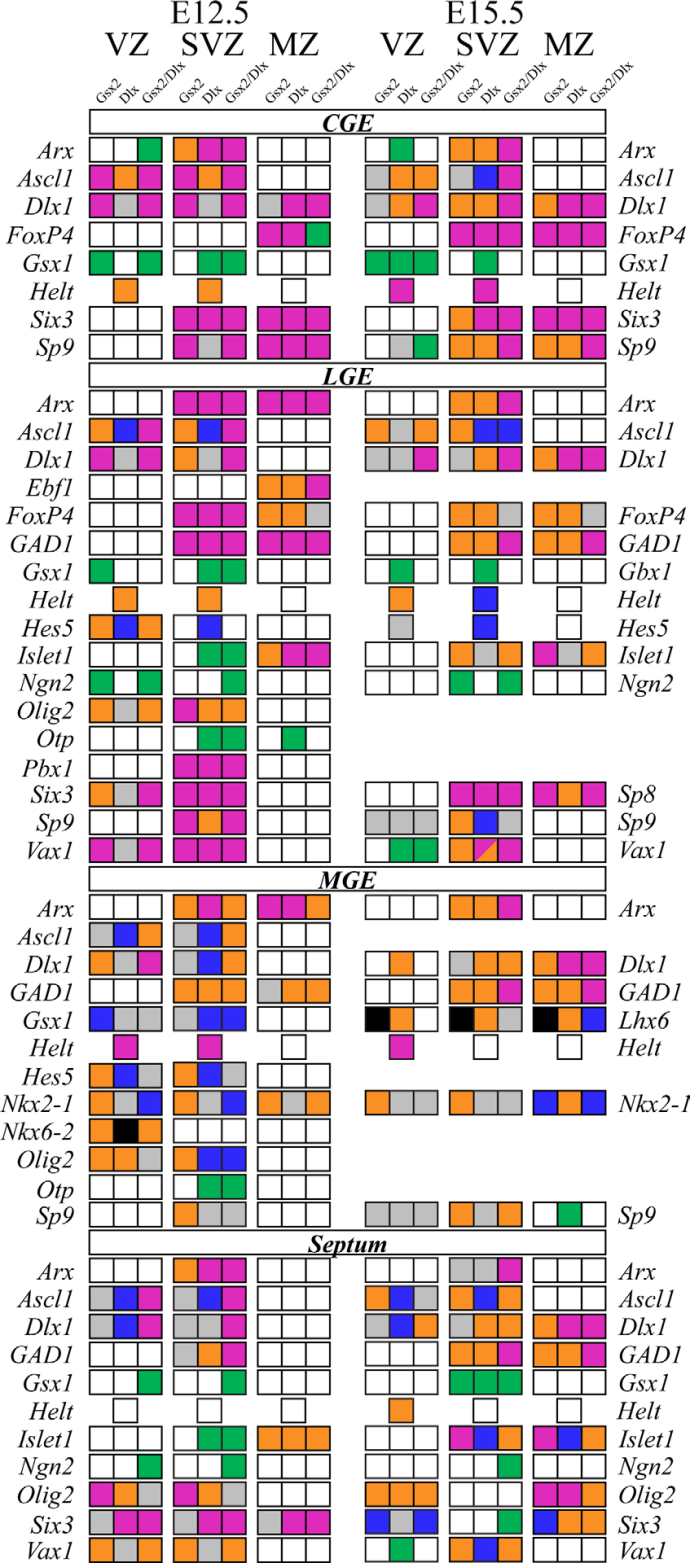
Expression of transcription factors in the ventricular zone (VZ), subventricular zone (SVZ), and mantle zone (MZ) of the LGE, MGE, and CGE in the Gsx2^−/−^ (Gsx2), Dlx1/2^−/−^ (Dlx), and Gsx2^−/−^;Dlx1/2^−/−^ (Gsx2/Dlx) mutants at E12.5 and E15.5. This figures depicts, as discrete boxes, the VZ, SVZ, and MZ of the CGE, LGE, MGE, and septum. The genes are listed alphabetically. The effect of each mutation on transcription factor expression in each box is indicated using a color code. Black indicates that expression was not analyzed (if no squares are listed, this also means that this analysis was not performed). Gray indicates that expression was not clearly changed in the mutant. White indicates no detectable expression. Red indicates severe reduction in expression. Orange indicates moderate/mild reduction in expression. Green indicates ectopic expression. Blue indicates increased expression. If the box is subdivided diagonally, the top part correspond to the dorsal region, the bottom to the ventral region.

**Figure 17:**
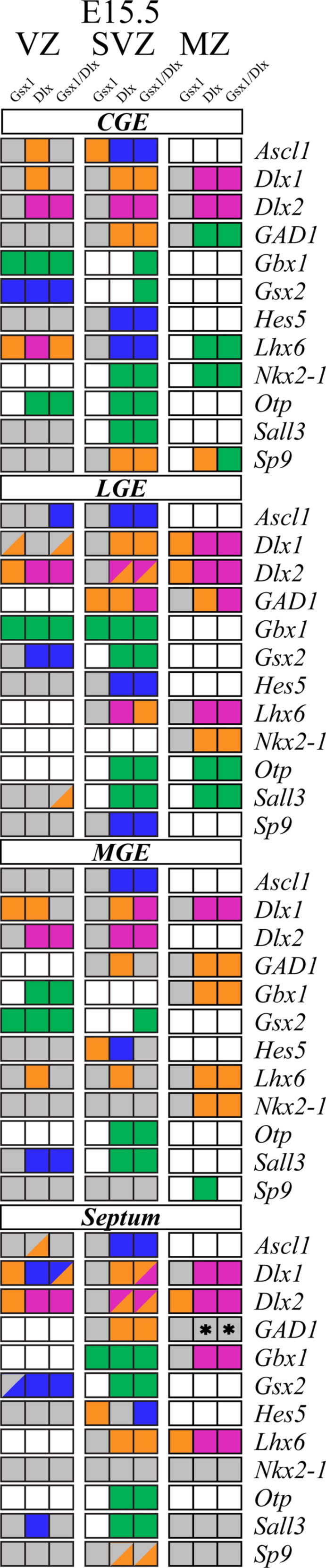
Expression of transcription factors in the ventricular zone (VZ), subventricular zone (SVZ), and mantle zone (MZ) of the LGE, MGE, and CGE in the Gsx1^−/−^ (Gsx1), Dlx1/2^−/−^ (Dlx), and Gsx1^−/−^;Dlx1/2^−/−^ (Gsx1/Dlx) mutants at E15.5. This figure depicts, as discrete boxes, the VZ, SVZ, and MZ of the CGE, LGE, MGE, and septum. The genes are listed alphabetically. The effect of each mutation on transcription factor expression in each box is indicated using a color code. Gray indicates that expression was not clearly changed in the mutant. White indicates no detectable expression. Red indicates severe reduction in expression. Orange indicates moderate/mild reduction in expression. Green indicates ectopic expression. Blue indicates increased expression. If no squares are listed, this means that this analysis was not performed. If the box is subdivided diagonally, the top part correspond to the dorsal region, the bottom to the ventral region. Asterisk indicates that diagonal band GAD1 expression was absent.

**Figure 18:**
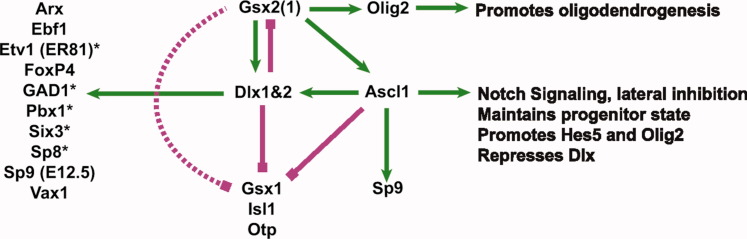
Model of transcription factor network interactions in the developing LGE based largely on loss-of-function analyses (see Discussion). Green arrows indicate activation; magenta squares indicate inhibition. Genes activated by Dlx1 and -2 that have asterisks correspond to genes whose expression is most strongly reduced in the Dlx1/2^−/−^ mutant. At the top of the hierarchy is Gsx2; Gsx2(1) indicates that Gsx1 can compensate for loss of Gsx2. For comparison see Long et al. (2009a,b) for summaries of gene expression changes in the Dlx1/2, Ascl1, and Dlx1/2;Ascl1 mutants, using the same schemata.

Elucidating transcriptional networks will be required to understand the mechanisms that control brain development. Here we have focused on the roles of the Gsx1 and -2 and Dlx1 and -2 genes in regulating the expression of 23 transcription factors during patterning and differentiation of the mouse subcortical telencephalon. Based on these results, and in previous publications on basal ganglia phenotypes in Dlx1 and -2 (Anderson et al., 1997b; Petryniak et al., 2007; Long et al., 2009a,b), Gsx1 and -2 (Corbin et al., 2000; Toresson et al, 2000; Toresson and Campbell, 2001; Yun et al., 2001, 2003; Waclaw et al., 2004), Ascl1 (Mash1; Casarosa et al., 1999; Horton et al., 1999; Yun et al., 2002; Castro et al., 2006; Long et al., 2009a,b), and Olig2 (Petryinak et al., 2007) mouse mutants, we have generated a provisional model of the genetic hierarchy of transcription factor genes in the LGE/dCGE (Fig. 18); a definitive model will require additional data, including demonstration of direct transcription regulation at each step. Here we address the basis for this model. We have summarized the gene expression changes in Figures 16 and 17; for comparison see Long et al. (2009a,b) for summaries of gene expression changes in the Dlx1/2, Ascl1, and Dlx1/2;Ascl1 mutants, using the same schemata.

### GSX2, ASCL1 (MASH1), and DLX2 expressions define their temporal hierarchy in the LGE

ASCL1 and DLX2 proteins are strongly expressed throughout the subpallium, but GSX2 expression is most easily detected in the LGE, septum, and CGE, although GSX2 is also expressed in the MGE. Here were focused on LGE expression at E10.5–E15.5 (Fig. 1, and not shown). Double-immunofluorescence analysis of GSX2, ASCL1, and DLX2 protein expression in the LGE provides evidence for a temporal hierarchy of their expression. At E10.5, the most immature cells (VZ cells) express only GSX2. As the VZ cells mature, scattered cells express ASCL1 and DLX2, most of which coexpress GSX2. By E12.5, many LGE progenitors (VZ + SVZ) coexpress GSX2, ASCL1, and DLX2 (Table 1). Coexpression is strongest in SVZ1, the part of the SVZ adjacent to the VZ.

The Dlx1/2;Ascl1 mutant phenotype of subpallial progenitors and neurons showed much more severe defects than either the Dlx1/2 or the Ascl1 mutants (Long et al., 2009a,b). Likewise, Gsx2 and Ascl1 double mutants showed more severe defects than the single mutants (Wang et al., 2009). Here we demonstrated a functional interaction between Gsx1 and Gsx2 with Dlx1/2 and provided evidence for the functional hierarchy of Gsx2, Gsx1, Ascl1, and Dlx1/2. We suggest that these phenotypes are due in large part to cell autonomous defects, particularly in the SVZ1, where GSX2, ASCL1, and DLX2 are coexpressed.

### Gsx2 homeodomain: top of the hierarchy of dLGE/dCGE identity

We propose that Gsx2 promotes the identity of primary progenitors in the VZ of the dLGE and dCGE. Gsx2 null mutants fail to specify dorsal parts of the LGE and CGE, showing reduced expression of other transcription factors that mark the VZ of these regions (Ascl1, Dlx2, Olig2). Our loss-of-function analysis is consistent with ectopic expression experiments in which cortical misexpression of Gsx2 induces Ascl1 and Dlx1/2 (Waclaw et al., 2009). Therefore, we hypothesize that Gsx2 promotes the expression of Ascl1, Dlx2, and Olig2, from which emanate three major pathways (Fig. 18): 1) neural differentiation driven by Dlx1 and -2; 2) lateral inhibition to promote the maintenance of multipotent progenitors driven by Ascl1 promoting Delta expression, which in turn increases Notch signaling and Hes5 expression; and 3) progenitor cell maintenance through Hes5 and competence to produce oligodendrocytes through Olig2.

### Gsx1 homedomain is upregulated in the absence of Gsx2 and Dlx1/2 and contributes to MGE phenotypes in the Dlx1/2 mutants

Previous studies showed that Gsx1 mutants have a very mild telencephalic phenotype. They have increased Gsx2 expression (Pei et al., 2011) and ectopic expression of Dbx1, a marker of the ventral cortex and preoptic area; the ramifications of this are not known. Gsx2 mutants are partially rescued by Gsx1, providing evidence that Gsx1 can compensate for Gsx2. Combined removal of Gsx1 and -2 leads to misspecification of the dorsal and ventral LGE (Toresson and Campbell, 2001; Yun et al., 2003). Overexpression of Gsx1 throughout the telencephalon (tetO-Gsx1-IRES-EGFP;Foxg1tTA/+ mice) induced Ascl1 and Dlx1/2. This result is similar to the phenotype of Gsx2 overexpression, confirming that Gsx1 and Gsx2 share properties (Pei et al., 2011). On the other hand, misexpressions of Gsx1 and Gsx2 show opposite effects on the switch between proliferation and differentiation: Gsx1 lengthens the cell cycle and promotes neurogenesis (and represses Gsx2 expression), whereas Gsx2 maintains the progenitor state (Waclaw et al., 2009; Pei et al., 2011). Thus, Gsx1 and Gsx2 share functions in promoting subpallial identity and appear to have opposite functions in regulating the switch between progenitor and neuronal fates.

Gsx2 and Dlx1 and -2 are negative regulators of Gsx1 (Fig. 1G–L; Toresson et al., 2000; Yun et al., 2001; Long et al., 2009a,b). Dlx1 and -2 repression of Gsx1 was explored here by making Gsx1;Dlx1/2 mutants. We found that loss of Gsx1 partially rescued MGE phenotypes in the Dlx1/2 mutant, including interneuron migration to the cortex (see below). As noted above, overexpression of Gsx1 promoted neurogenesis and repressed proliferation (Pei et al., 2011); therefore, we had anticipated that removing Gsx1 in the Dlx1/2 mutants might further block their differentiation. On the contrary, the MGE of the Gsx1;Dlx1/2 mutants had a subtle reduction of Hes5 expression (indicator of Notch signaling) compared with Dlx1/2 mutants and had increased GAD1 and Lhx6 expression (indicators of differentiation; Fig. 14). Thus, in the Dlx1/2 mutant MGE, removing Gsx1 reduced Hes5 (Notch signaling), which is similar to the effect of removing Gsx2 in the Dlx1/2 mutant (Fig. 4).

### Two functions of Ascl1 (Mash1) bHLH: promoting the subcortical progenitor state through Notch signaling and promoting subcortical differentiation with Gsx2 and Dlx1/2

Previous studies demonstrated that Ascl1 promotes the subcortical progenitor state through cell autonomously increasing Delta1 expression and cell nonautonomously (through lateral inhibition) increasing Notch signaling and repressing Dlx expression (Casarosa et al., 1999; Horton et al., 1999; Yun et al., 2002; Castro et al., 2006); this has the effect of repressing neurogenesis and promoting gliogenesis, including oligodendrogenesis (Parras et al., 2007; Petryniak et al. 2007). Ascl1 mutants continue to express Gsx2 at roughly normal levels at E12.5 (Wang et al., 2009) and E15.5 (Long and Rubenstein, unpublished).

Ascl1;Gsx2 compound mutants have a severe reduction in LGE differentiation (Wang et al., 2009), despite continued expression of Gsx1. Thus, Gsx2 and Ascl1 together contribute to specifying the LGE developmental program.

Analysis of Ascl1;Dlx1/2 individual and compound mutants provided evidence for distinct Ascl1- and Dlx1/2dependent pathways of LGE/dCGE development; we have proposed that the Ascl1 pathway operates through promoting the expression of Hes5, Olig2, and Sp9; the Dlx pathway components are described below (Long et al., 2007, 2009a,b).

Gsx2 also is a positive regulator of Ascl1, Hes5, and Sp9 (which could occur through Ascl1; Figs. 2, 6, 9; Wang et al., 2009). Thus, Gsx2 and Ascl1 share common regulatory functions for promoting Notch signaling (based on Hes5 expression) and Sp9 expression that distinguish them from Dlx1/2 function.

Ascl1;Dlx1/2 compound mutants have greatly reduced subcortical differentiation but continue to express limited aspects of subcortical identity, based on expression of GAD1 and truncated Ascl1 and Dlx1 RNAs; we have postulated that subcortical identity is maintained in these mutants through the function of a few key transcription factors, including Gsx1 and -2 and Islet1 (Long et al., 2009a,b). Thus, to evaluate the core functions of Gsx1 and -2, we generated the Gsx2;Dlx1/2 mutants and Gsx1;Dlx1/2 mutants.

### Elimination of Gsx2 partially rescues subpallial Notch signaling and oligodendrogenesis deficits in Dlx1/2 mutants

The Dlx genes promote LGE/dCGE development through controlling the expression of multiple transcription factors (Figs. 2, 4–6, 11; Long et al., 2009a,b). Generally, they repress the expression of transcription factors that promote the progenitor and/or glia cell state, including Ascl1, Gsx1 and -2, Hes5, and Olig2. The block in subcortical neural differentiation in Dlx1 and -2 mutants may be due, in part, to persistent expression of transcription factors that promote progenitor cell properties. For instance, in the Dlx1 and -2 mutants, there is overexpression of Olig2 that is linked to their overproduction of oligodendrocytes (Petryniak et al., 2007). This phenotype is reversed in Ascl1;Dlx1/2 compound mutants (Petryniak et al., 2007).

Compound Gsx2;Dlx1/2 mutants also show a recovery of specific aspects of the Dlx1/2 mutant phenotype. In the Gsx2;Dlx1/2 mutants, there was rescue of progenitor zone overexpression of several transcription factors, including Ascl1, Hes5, Olig2, and Gbx1 (Figs. 2–7, 11). The reduction in Ascl1 and Hes5 expression at E12.5 and E15.5 provides evidence that the increase in Notch signaling present in the Dlx1/2 mutants is mediated by Gsx2 expression. The reduction in Olig2 expression provides evidence that the Ascl1-mediated increase in oligodendrogensis present in the Dlx1/2 mutants (Petryniak et al., 2007) is mediated via Gsx2 promoting Ascl1 expression.

### Elimination of Gsx2 exacerbates LGE/dCGE specification and differentiation defects in Dlx1/2 mutants

Although certain features of the subpallial progenitors are partially rescued in the Gsx2;Dlx1/2 compound mutants, other features are worsened compared with the Gsx2 and Dlx1/2 mutants, including regional specification/patterning and neural differentiation. The LGE, CGE, and septum show regional patterning defects, including ectopic expression of pallial markers (Ngn2), loss of subpallial markers (Ascl1, Dlx1, Vax1), and frank hypoplasia, especially of the CGE (Figs. 2, 4, 6, 11). We postulate that reducing Ascl1 levels in the Gsx2;Dlx1/2 compound mutants is an important mechanism that contributes to their these phenotypes.

Many of the Gsx2;Dlx1/2 compound mutant's phenotypes are partially rescued in the LGE and septum by E15.5 (Figs. 3, 12), whereas the CGE continues to be extremely hypoplastic at this stage (Figs. 7. 10). Thus, as in the Gsx2 mutant, there is a time-dependent aspect to the LGE patterning defect (Corbin et al., 2000; Toresson et al., 2000; Yun et al., 2001); previous work demonstrated that upregulation of Gsx1 contributes to this temporal rescue (Toresson and Campbell, 2001; Yun et al., 2003).

In addition to exacerbating patterning defects, removal of Gsx2 function in the Dlx1/2 mutants increases their neural differentiation defects. Dlx1/2 promotes the expression of transcription factors that direct specific pathways of neural differentiation, including Arx, Dlx5 and -6, EBF, Pbx1, Six3, Sp8, and Vax1, as well as expression of key features of the GABAergic state, including GAD1 expression (Long et al., 2007, 2009a,b).

Gsx2;Dlx1/2 compound mutants have further reductions in the expression of critical regulators of subpallial neuronal development, including Arx, GAD1, and Islet1 (Figs. 5, 9, 10, 12). Notably, although GAD1 expression is maintained in the septum and MGE of the Dlx1/2 mutants, its expression is nearly lost throughout the subpallium of the E15.5 Gsx2;Dlx1/2 compound mutants (Figs. 10, 12). GAD1 expression is present, albeit reduced, at E12.5 (Figs. 5, 9). Thus, together, Dlx1/2 and Gsx2 are essential for promoting and maintaining expression of GAD1, a central feature of forebrain GABAergic neurons.

### Elimination of Gsx1 partially rescues Dlx1/2 mutant MGE properties, including interneuron migration

Whereas Gsx, Mash, and Dlx participate in MGE differentiation, regional and cellular fate specification in this region also operates through a parallel/overlapping program mediated by the Nkx2.1 and the Lhx6/7(8) genes (Sussel et al., 1999; Liodis et al., 2007; Zhao et al., 2008; Flandin et al., 2011). This may explain why MGE properties are relatively better preserved than LGE/CGE/septal properties in the Gsx2;Dlx1/2 mutants. For instance, at E12.5 Arx, GAD1, and Sp9 expression are maintained only in the MGE (Fig. 9). We suggest that the preserved expression of Ascl1 and Nkx2.1 plays a major role in maintaining the MGE developmental program in the Gsx2;Dlx1/2 mutants.

Gsx1 appears to be more important than Gsx2 in MGE development. Remarkably, the Gsx1;Dlx1/2 mutants, but not the Gsx2;Dlx1/2 mutants, show a partial rescue of the migration of Lhx6^+^ cells to the cortex (Figs. 13–15). Loss of Gsx1 normalizes molecular properties of the Dlx1/2 mutant MGE, including increasing Lhx6 and GAD1 expression and reducing Hes5 expression (Notch signaling marker; Fig. 14). Thus, Gsx1 overexpression in the MGE may repress its differentiation, including its ability to produce interneurons that can migrate to the cortex. Alternatively, Gsx1 overexpression may alter the expression of factors that directly promote interneuron migration. In either case, overexpression of Gsx1 contributes to the block of interneuron migration of the Dlx1/2 mutants. It should be noted that, despite the partial rescue, most MGE-derived interneurons in the Gsx1;Dlx1/2 mutants remain in the subpallium, as in the Dlx1/2 mutant. Many of these cells appear to coalesce as an ectopia in the caudoventral subpallium, which expresses Gad1, Lhx6, Nkx2.1, and Sp9 (Fig. 15).

### Independent and combined functions of Gsx1 and Gsx2, with Dlx1/2, in septal development

Septal and LGE development share many similarities, but a key difference in the transcription programs of the LGE and the septum is that septal expression of Dlx5/6 is preserved in the Dlx1/2 mutant (Long et al., 2009a). Furthermore, the septum and the ventral LGE are particularly sensitive to loss of Ascl1 function (Long et al., 2009a).

Our previous and current analyses of the Ascl1 and Dlx1/2 mutants revealed, for instance, that Dlx1 and -2 are positive regulators of ER81, Gbx1, Pbx1, and Six3, whereas Ascl1 promotes expression of Arx, Hes5, Islet1, Olig2, Sp9, and Vax1 (Long et al., 2009a; Figs. 11, 12). Here we extend our analysis of the Dlx1/2 mutants; the results are summarized in Figures 16 and 17.

This is the first analysis of septal development in Gsx2 and Gsx1 mutants. We have provided evidence that Gsx2 is required for expression of Ascl1, FoxP4, Islet1, Olig2, Sp9, and Vax1 (Figs. 11, 12, 16, 17). We propose, based on these findings, that in septal progenitors Gsx2 lies upstream of both Ascl1 and Dlx1/2. Loss of either Gsx2 or Ascl1 leads to reduced expression of Islet1, Olig2, Sp9, and Vax1, whereas loss of Dlx1/2 leads to increased expression of these genes (Fig. 11). The septal phenotype in the Gsx2;Dlx1/2 compound mutant was more severe than in either the Gsx2 or the Dlx1/2 mutant; the septum had reduced expression of supallial properties (Ascl1, Dlx1, and GAD1) and increased expression of the pallial marker (Ngn2), even through E15.5 (Figs. 11, 12). Thus, together, Gsx2 and Dlx1/2 are required for septal development. Gsx2 drives the Ascl1 program (Islet1, Olig2, Sp9, and Vax1), and Dlx1/2 drives a parallel program (ER81, Gbx1, Pbx1, and Six3).

Gsx1 also regulates septal development, particularly in the medial septum, where Dlx1, Gbx1, and Lhx6 expression are reduced (Fig. 13). The medial septal domain is generated from the MGE region (Flandin et al., 2010), consistent with Gsx1's MGE expression. Removing Gsx1 expression in the Dlx1/2 mutants showed a subtle rescue of septal and CGE phenotypes, including expression of Ascl1, Dlx1, and Sall3, showing that Gsx1 upregulation in the Dlx1/2 mutant septum contributes to its phenotypes (Figs. 13, 16, 17).

## ROLE OF AUTHORS

All authors had full access to all the data in the study and take responsibility for the integrity of the data and the accuracy of the data analysis. Study concept and design: JLRR. Acquisition of data: BW, PF, JEL, RRW. Analysis and interpretation of data: BW, PF, JEL, RP, RRW, KC, JLRR. Drafting of the manuscript: BW, PF, JEL, RRW, KC, JLRR. Critical revision of the manuscript for important intellectual content: BW, PF, JEL, RRW, KC, JLRR. Statistical analysis: RP. Obtained funding: KC, JLRR. Administrative, technical, and material support: BW, PF, JEL, RRW. Study supervision: KC, JLRR.
